# Artificial intelligence–based, volumetric assessment of the bone marrow metabolic activity in [^18^F]FDG PET/CT predicts survival in multiple myeloma

**DOI:** 10.1007/s00259-024-06668-z

**Published:** 2024-03-08

**Authors:** Christos Sachpekidis, Olof Enqvist, Johannes Ulén, Annette Kopp-Schneider, Leyun Pan, Elias K. Mai, Marina Hajiyianni, Maximilian Merz, Marc S. Raab, Anna Jauch, Hartmut Goldschmidt, Lars Edenbrandt, Antonia Dimitrakopoulou-Strauss

**Affiliations:** 1 Clinical Cooperation Unit Nuclear Medicine, German Cancer Research Center (DKFZ), Im Neuenheimer Feld 280, 69210 Heidelberg, Germany; 2 Eigenvision AB, Malmö, Sweden; 3 Department of Electrical Engineering, Chalmers University of Technology, Gothenburg, Sweden; 4 Division of Biostatistics, German Cancer Research Center (DKFZ), Heidelberg, Germany; 5 Department of Internal Medicine V, University Hospital Heidelberg and National Center for Tumor Diseases (NCT), Heidelberg, Germany; 6 Department of Hematology and Cell Therapy, University of Leipzig, Leipzig, Germany; 7 Institute of Human Genetics, University of Heidelberg, Heidelberg, Germany; 8 Department of Clinical Physiology, Region Västra Götaland, Sahlgrenska University Hospital, Gothenburg, Sweden; 9 Department of Molecular and Clinical Medicine, Institute of Medicine, Sahlgrenska Academy, University of Gothenburg, Gothenburg, Sweden

**Keywords:** Multiple myeloma, Prognosis, Patient survival, [^18^F]FDG PET/CT, Deep learning, Artificial intelligence, Metabolic tumor volume (MTV), Total lesion glycolysis (TLG)

## Abstract

**Purpose:**

Multiple myeloma (MM) is a highly heterogeneous disease with wide variations in patient outcome. [^18^F]FDG PET/CT can provide prognostic information in MM, but it is hampered by issues regarding standardization of scan interpretation. Our group has recently demonstrated the feasibility of automated, volumetric assessment of bone marrow (BM) metabolic activity on PET/CT using a novel artificial intelligence (AI)–based tool. Accordingly, the aim of the current study is to investigate the prognostic role of whole-body calculations of BM metabolism in patients with newly diagnosed MM using this AI tool.

**Materials and methods:**

Forty-four, previously untreated MM patients underwent whole-body [^18^F]FDG PET/CT. Automated PET/CT image segmentation and volumetric quantification of BM metabolism were based on an initial CT-based segmentation of the skeleton, its transfer to the standardized uptake value (SUV) PET images, subsequent application of different SUV thresholds, and refinement of the resulting regions using postprocessing. In the present analysis, ten different uptake thresholds (AI approaches), based on reference organs or absolute SUV values, were applied for definition of pathological tracer uptake and subsequent calculation of the whole-body metabolic tumor volume (MTV) and total lesion glycolysis (TLG). Correlation analysis was performed between the automated PET values and histopathological results of the BM as well as patients’ progression-free survival (PFS) and overall survival (OS). Receiver operating characteristic (ROC) curve analysis was used to investigate the discrimination performance of MTV and TLG for prediction of 2-year PFS. The prognostic performance of the new Italian Myeloma criteria for PET Use (IMPeTUs) was also investigated.

**Results:**

Median follow-up [95% CI] of the patient cohort was 110 months [105–123 months]. AI-based BM segmentation and calculation of MTV and TLG were feasible in all patients. A significant, positive, moderate correlation was observed between the automated quantitative whole-body PET/CT parameters, MTV and TLG, and BM plasma cell infiltration for all ten [^18^F]FDG uptake thresholds. With regard to PFS, univariable analysis for both MTV and TLG predicted patient outcome reasonably well for all AI approaches. Adjusting for cytogenetic abnormalities and BM plasma cell infiltration rate, multivariable analysis also showed prognostic significance for high MTV, which defined pathological [^18^F]FDG uptake in the BM via the liver. In terms of OS, univariable and multivariable analysis showed that whole-body MTV, again mainly using liver uptake as reference, was significantly associated with shorter survival. In line with these findings, ROC curve analysis showed that MTV and TLG, assessed using liver-based cut-offs, could predict 2-year PFS rates. The application of IMPeTUs showed that the number of focal hypermetabolic BM lesions and extramedullary disease had an adverse effect on PFS.

**Conclusions:**

The AI-based, whole-body calculations of BM metabolism via the parameters MTV and TLG not only correlate with the degree of BM plasma cell infiltration, but also predict patient survival in MM. In particular, the parameter MTV, using the liver uptake as reference for BM segmentation, provides solid prognostic information for disease progression. In addition to highlighting the prognostic significance of automated, global volumetric estimation of metabolic tumor burden, these data open up new perspectives towards solving the complex problem of interpreting PET scans in MM with a simple, fast, and robust method that is not affected by operator-dependent interventions.

**Supplementary Information:**

The online version contains supplementary material available at 10.1007/s00259-024-06668-z.

## Introduction

Multiple myeloma (MM) is considered an incurable hematological malignancy. The duration and quality of life for patients continue to improve [1]. At the same time, MM is a very heterogeneous disease with wide variations in clinical course and outcome among patients, largely due to the fact that plasma cells display distinct malignant potential, e.g., based on their cytogenetic profile or genetic alterations [2]. The prognosis and patient survival in MM are affected by multiple factors, among which tumor burden plays a key role [3]. Tumor burden is estimated using the Durie-Salmon staging [4] and the International Staging System (ISS) [5], and nowadays the Revised International Staging System (R-ISS) [6].

For an accurate individual assessment of the extent of the disease and its prognosis, imaging has evolved into a key component. Among cross-sectional imaging modalities, which have replaced skeletal radiography, traditionally used in the Durie and Salmon system, [^18^F]FDG PET/CT is gradually gaining in importance. Performed at the initial diagnosis of MM, [^18^F]FDG PET/CT has been shown to provide significant prognostic information based on the presence and number of focal lesions and diffuse bone marrow (BM) infiltration [7–10]. In addition, [^18^F]FDG PET/CT is considered to be the most appropriate method for assessing treatment response in this disease because of its ability to reliably distinguish metabolically active from inactive myeloma lesions [8, 9].

However, [^18^F]FDG PET/CT does have certain limitations concerning individual MM evaluation. Due to the different patterns of BM involvement as well as the non-negligible incidence of concomitant, myeloma-related events of adverse prognostic impact, such as the development of paramedullary disease (PMD), extramedullary disease (EMD), and pathological fractures, the standardization of assessment and reporting of the PET/CT examinations may be difficult to obtain, which, in turn, negatively affects inter-observer reproducibility in scan interpretation [11, 12].

One approach to address the issue of standardization of [^18^F]FDG PET/CT evaluation involves the volumetric assessment of the metabolic activity in the whole BM compartment—mainly through the calculation of the parameters metabolic tumor volume (MTV) and total lesion glycolysis (TLG)—which incorporates information both on focal myeloma lesions and diffuse BM infiltration [13, 14]. However, the accurate calculation of these volumetric parameters may prove to be a challenging task, requiring considerable computing power and fast and reproducible computer programs to allow for proper segmentation and correction of background activity and partial volume effect [15].

Our group has recently validated a novel artificial intelligence (AI)–based tool for whole-body volumetric assessment of BM metabolic activity on PET/CT images in MM. We demonstrated that automated BM segmentation and calculation of MTV and TLG are feasible, while AI-derived quantitative PET parameters significantly correlate with the results of visual analysis of scans as well as with the biopsy-derived BM plasma cell infiltration rate, highlighting the potential role of deep learning methods towards optimization and standardization of PET/CT interpretation [16].

In this context, and in an attempt to deepen the existing knowledge on the perspectives of AI in PET/CT for MM diagnostics, we investigated the prognostic role of automatically obtained whole-body volumetric calculations of BM metabolism in patients with newly diagnosed MM in the current study. In addition, the study aimed to identify potential [^18^F]FDG uptake thresholds for effective BM segmentation in terms of patient prognosis.

## Materials and methods

### Patients

Forty-four patients (29 male, 15 female; mean age 58.2 years) with previously untreated MM based on the criteria established by the International Myeloma Working Group (2003) were included in this analysis (Table 1) [17]. All patients received bortezomib-based induction therapy followed by high-dose chemotherapy (HDT) and autologous stem cell transplantation (ASCT). Twenty-one patients were enrolled in the prospective, open-label, randomized GMMG-MM5 phase III trial (EudraCT No. 2010–019173-16), which compared two different bortezomib-based induction regimens, followed by HDT, ASCT, and lenalidomide maintenance for 2 years or until complete response [18], while the remaining 23 patients were treated with comparable regimens and ASCT outside the trial. Patients of this cohort were previously studied by our group in other analyses but with different approaches and shorter follow-up as presented here [19, 20]. No patient had received chemotherapy, granulocyte colony-stimulating factor (G-CSF), or erythropoietin before the study inclusion. The study was conducted in accordance with the Declaration of Helsinki principles, with institutional approval by the ethical committee of the University of Heidelberg (S-076/2010) and the Federal Agency of Radiation Protection in Germany (“Bundesamt für Strahlenschutz”). All patients gave written informed consent prior to participation in this study.

**Table 1:** Patient characteristics (*N* = 44)

Patient characteristics	Value
Median age, years	60 (38–73)
Sex	
Male	29 (66%)
Female	15 (34%)
Median albumin, g/dL	39.8 (17.7–50.2)
Median β2-microglobulin, mg/L	3.0 (1.1–17.0)
Median LDH, μ/L	185 (123–300)
Median bone marrow plasma cell infiltration	38% (1–92%)
High-risk cytogenetics	
Yes	8 (18%)
No	31 (70%)
Unknown	5 (11%)
ISS stage	
I	23 (52%)
II	13 (30%)
III	4 (9%)
Not defined	4 (9%)
R-ISS stage	
I	13 (30%)
II	20 (45%)
III	2 (5%)
Not defined	9 (20%)
Second-line therapies after first disease relapse/progression	
PI-based	4
IMiD-based	5
Mab-based	2
Mab- and PI-based	2
Mab- and IMiD-based	1
Cytotoxic agent–based	6
PI- and IMiD-based	4
Re-induction + HDT + ASCT	7
Other	2
No treatment required	11

*LDH*, lactate dehydrogenase; *PI*, proteasome inhibitor; *IMiD*, immunomodulatory drug; *Mab*, monoclonal antibody; *HDT*, high-dose chemotherapy; *ASCT*, autologous stem cell transplantation

### PET/CT data acquisition

All patients underwent whole-body [^18^F]FDG PET/CT at diagnosis before commencement of treatment. Imaging was performed 60 min after injection of the radiopharmaceutical from the skull to the toes with a scan duration of 2 min per bed position. A dedicated PET/CT system (Biograph mCT, S128, Siemens Co., Erlangen, Germany) with an axial field of view of 21.6 cm with TruePoint and TrueV operated in a three-dimensional mode was used. A low-dose attenuation CT (120 kV, 30 eff mA) was used for attenuation correction of the PET data and for image fusion. All PET images were attenuation-corrected and an image matrix of 400 × 400 pixels was used for iterative image reconstruction. Iterative image reconstruction was based on the ordered subset expectation maximization (OSEM) algorithm with two iterations and 21 subsets as well as time of flight (TOF).

### PET/CT data analysis

#### Automated PET/CT data analysis and quantification

Automated [^18^F]FDG PET/CT image segmentation and volumetric quantification were performed as described previously [16]. Briefly, the applied deep learning–based methodology consists of the following three steps:

1. CT-based organ segmentation: With the use of a convolutional neural network [21], segmentation of the skeleton, the liver, and muscles was performed. The bones were divided into *bones of the axial skeleton*, including the vertebrae, scapulae, clavicles, sternum, ribs, sacrum, and pelvic bones, and into *bones of the extremities*, including the humeri and femora. In order to avoid the effect of the intense physiological [^18^F]FDG uptake from the brain, the skull was excluded frοm the evaluations.
2. Application of standardized uptake value (SUV) threshold(s) in the relevant organs: The CT-based segmentation was transferred to the SUV PET images, and then, different SUV thresholds were applied to identify BM infiltration. All pixels with SUV above or equal to the threshold were segmented as positive for MM infiltration, after employment of specific steps to mitigate the effect of potential spillover of tracer uptake from adjacent tissues into the bone mask due to the poor resolution of PET images [16]. In the present study, ten different SUV thresholds were employed to define pathological [^18^F]FDG uptake in the skeleton. Four of these thresholds had already been tested in the initial application of the tool in MM where they showed a significant correlation with the BM plasma cell infiltration rate (*approaches 1–4*) [16]. The remaining six were developed based on modifications of the previous approaches with the aim of optimizing or complementing the existing thresholds (*approaches 5–10*). In particular, the thresholds applied were the following:
  *Approach 1* Axial skeleton: SUV ≥ liver SUVmedian × 1.1. Extremities: SUV ≥ muscle SUVmedian × 4.
  *Approach 2* Axial skeleton: SUV ≥ liver SUVmedian × 1.5. Extremities: SUV ≥ muscle SUVmedian × 4.
  *Approach 3* Axial skeleton and extremities: SUV ≥ 2.5, according to Terao et al. [14].
  *Approach 4* Axial skeleton: SUV ≥ 2.5. Extremities: SUV ≥ 2.0.
  *Approach 5* Axial skeleton and extremities: SUV ≥ liver SUVmedian.
  *Approach 6* Axial skeleton: SUV ≥ liver SUVmedian. Extremities: SUV ≥ muscle SUVmedian × 4.
  *Approach 7* Axial skeleton and extremities: SUV ≥ liver SUVmedian × 1.2.
  *Approach 8* Axial skeleton: SUV ≥ liver SUVmedian × 1.2. Extremities: SUV ≥ muscle SUVmedian × 4.
  *Approach 9* Axial skeleton and extremities: SUV ≥ liver SUVmean.
  *Approach 10* Axial skeleton and extremities: SUV ≥ 3.0 (Table 2).
3. Refinement of the resulting regions using postprocessing and subsequent calculation of whole-body MTV and TLG: Using the resulting masks, the total whole-body MTV could be estimated as the volume of the segmented pathological uptake in each patient. In particular, MTV (mL) represents the volume of myeloma lesions visualized on PET/CT with SUV greater than a predefined threshold. Similarly, TLG was calculated as the product of average SUV and MTV for the segmented regions (TLG = SUVmean × MTV).

**Table 2:** The different SUV thresholds applied for definition of pathological tracer uptake in the BM with the AI tool, their median values (range) in the studied cohort, and the respective *r* and *p*-values of the correlation analysis between MTV, TLG, and the BM infiltration rate by malignant plasma cells

AI approaches	Applied threshold	Median MTV (range), mL	Median TLG (range), g	Correlation with BM infiltration rate
Approach 1	*Axial skeleton*: liver SUVmedian × 1.1 *Extremities*: muscle SUVmedian × 4	203.4 (11.2–1161.3)	591.6 (31.0–4339.2)	*r* = 0.46, *p* < 0.01 (MTV)* *r* = 0.43, *p* < 0.01 (TLG)*
Approach 2	*Axial skeleton*: liver SUVmedian × 1.5 *Extremities*: muscle SUVmedian × 4	26.9 (0–608.7)	95.5 (0–2719.3)	*r* = 0.39, *p* < 0.01 (MTV)* *r* = 0.38, *p* = 0.01 (TLG)*
Approach 3	*2.5*	104.6 (0–1133.6)	327.9 (0–4268.2)	*r* = 0.35, *p* = 0.02 (MTV)* *r* = 0.34, *p* = 0.02 (TLG)*
Approach 4	*Axial skeleton*: 2.5 *Extremities*: 2.0	111.8 (0–1219.7)	366.3 (0–4464.8)	*r* = 0.34, *p* = 0.02 (MTV)* *r* = 0.34, *p* = 0.02 (TLG)*
Approach 5	liver SUVmedian	346.9 (33.9–1446.0)	884.2 (87.3–5022.4)	*r* = 0.44, *p* < 0.01 (MTV)* *r* = 0.41, *p* < 0.01 (TLG)*
Approach 6	*Axial skeleton*: liver SUVmedian *Extremities*: muscle SUVmedian × 4	329.4 (33.9–1386.5)	849.2 (87.3–4875.0)	*r* = 0.46, *p* < 0.01 (MTV)* *r* = 0.42, *p* < 0.01 (TLG)*
Approach 7	liver SUVmedian × 1.2	126.8 (0–974.2)	396.5 (0–3844.1)	*r* = 0.45, *p* < 0.01 (MTV)* *r* = 0.42, *p* < 0.01 (TLG)*
Approach 8	*Axial skeleton*: liver SUVmedian × 1.2 *Extremities*: muscle SUVmedian × 4	126.2 (0–975.3)	398.1 (0–3847.1)	*r* = 0.46, *p* < 0.01 (MTV)* *r* = 0.43, *p* < 0.01 (TLG)*
Approach 9	Liver SUVmean	314.3 (29.1–1404.0)	836.9 (75.8–4926.4)	*r* = 0.44, *p* < 0.01 (MTV)* *r* = 0.43, *p* < 0.01 (TLG)*
Approach 10	3.0	26.5 (0–739.7)	118.9 (0–3157.5)	*r* = 0.34, *p* = 0.03 (MTV)* *r* = 0.32, *p* = 0.04 (TLG)*

^*^Statistically significant correlation (*p* < 0.05)

#### Application of IMPeTUs

Two nuclear medicine physicians (first and last authors) independently analyzed the [^18^F]FDG PET/CT images on an Aycan workstation. Disagreements between the readers were resolved by consensus. Image interpretation was based on IMPeTUs [11], which briefly consider the following parameters:

- BM metabolic state calculated in the lower lumbar spine in the absence of focal tracer enhancement, based on the 5-point Deauville score (DS): score 1, no uptake at all; score 2, ≤ mediastinal blood pool uptake; score 3, > mediastinal blood pool uptake, ≤ liver uptake; score 4, > liver uptake + 10%; score 5, >  > liver uptake (twice).
- Number of focal, [^18^F]FDG-avid medullary lesions, consistent with MM (Fx): F1, no lesions; F2, 1–3 lesions; F3, 4–10 lesions; F4, > 10 lesions.
- Location of focal, [^18^F]FDG-avid medullary lesions, consistent with MM: skull, spine, other.
- Degree of [^18^F]FDG uptake of the hottest MM lesion, based on DS score: 1, no uptake at all; score 2, ≤ mediastinal blood pool uptake; score 3, > mediastinal blood pool uptake, ≤ liver uptake; score 4, > liver uptake + 10%; score 5, >  > liver uptake (twice).
- Number of lytic lesions on CT (Lx): L1, no lesions; L2, 1–3 lesions; L3, 4–10 lesions; L4, > 10 lesions.
- Presence of at least one fracture on CT.
- Presence of PMD, defined as a hypermetabolic bone lesion extending through the cortical bone and involving the surrounding soft tissues.
- Presence of EMD, defined as hypermetabolic myeloma lesion in the soft tissues without bone involvement.

### Clinical parameters, BM plasma cell infiltration, and fluorescence in situ hybridization

BM aspirates or biopsies were performed within 4 weeks of the [^18^F]FDG PET/CT examination and prior commencement of treatment. The routine BM biopsy procedure in our center involves collection of 1–3-cm-long BM core taken from the iliac crest. Percentage of BM infiltration by malignant plasma cells was assessed via Giemsa-stained bone marrow smears. The infiltration rate is the ratio of the number of plasma cells to the number of all nucleated cells in BM. Fluorescence in situ hybridization was performed, as described previously [22] on CD138-purified plasma cells using the following probes: 1q21, 5p15, 5q35, 8p21, 9q34, 11q22.3, 13q14, 15q22, 17p13, and 19q13. We also investigated immunoglobulin H (IgH) translocations using an IgH break-apart probe as well as probes for t(11;14), t(4;14), and t(14;16). The R-ISS score was used to define high-risk disease. According to this prognostic system, stage R-ISS I included patients of ISS stage I (serum β2-microglobulin level < 3.5 mg/L and serum albumin level ≥ 3.5 g/dL); no high-risk chromosomal abnormalities, defined as deletion 17p13, and/or translocation t(4;14) and/or translocation t(14;16); and normal lactate dehydrogenase (LDH) level; stage R-ISS III included ISS stage III (serum β2-microglobulin level > 5.5 mg/L) and high-risk chromosomal abnormalities or high LDH level; stage R-ISS II included all the other possible combinations [6].

### Statistical analysis

For all approaches, MTV and TLG measurements showed a skewed distribution. Therefore, median and range values are reported. Consequently, correlation analysis of MTV and TLG measurements with BM infiltration rate was based on Spearman rank correlation. Progression-free survival (PFS) was defined as time from PET/CT to disease progression or death from any cause, whichever occurs first, and overall survival (OS) was defined as the time from PET/CT until death from any cause or last follow-up. Kaplan–Meier estimates were generated and median PFS and OS estimated. Median follow-up time was determined by inverse Kaplan–Meier estimation. For univariable comparison of PFS and OS, log-rank test was used, dichotomizing the quantitative variables at their median. Univariable Cox proportional hazards regression analysis was applied for the MTV and TLG measurements and parameters included in IMPeTUs. Multivariable Cox proportional hazards regression analysis was applied to investigate association between survival time of patients and multiple predictors simultaneously. For parameters highly correlated with each other, such as MTV and TLG, only one was included at a time in the model. No correction for multiple testing was performed as the study was exploratory. The receiver operating characteristic (ROC) curve was used to investigate the discrimination performance of MTV and TLG for prediction of survival in the first 2 years after PET/CT. The area under the curve (AUC) was calculated, and the cut point optimizing the Youden index, i.e., the sum of sensitivity and specificity, was determined for each approach. The results were considered significant for *p*-value less than 0.05 (*p* < 0.05). Calculations were performed with R version 4.1.1. and R packages survival, survminer, prodlim, timeROC, and pROC (R Foundation for Statistical Computing, Vienna, Austria; https://www.R-project.org/).

## Results

### Patient characteristics

The median BM plasma cell infiltration rate ranged between 1 and 92%, with a mean value of 41% (median 38%). Twenty-three patients were classified as ISS stage I, 13 patients as ISS stage II, and four patients as ISS stage III. Cytogenetic data were available for 39 patients, with high-risk cytogenetic abnormalities being detected in eight of them. A combination of the ISS and cytogenetic data was available in 35 patients. Based on this, 13 patients were classified as R-ISS-I group, 20 patients as R-ISS-II group, and two patients as R-ISS-III group. The patients’ characteristics at the time of PET/CT as well as data on the second line of treatment after bortezomib induction, ASCT, and lenalidomide maintenance in patients with relapse/progression are summarized in Table 1.

### Automated [^18^F]FDG PET/CT quantification

**Fig. 1:**
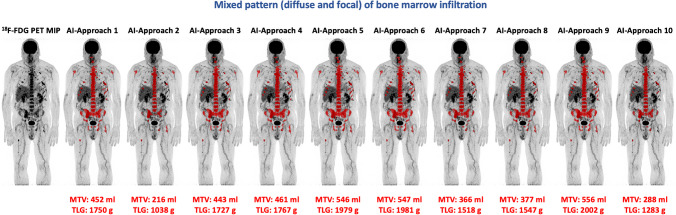
Example of the application of the AI-based software tool for automated calculation of total MTV and TLG of a MM patient with intense diffuse BM [^18^F]FDG uptake as well as multiple focal hypermetabolic lesions. The use of different tracer uptake thresholds leads to different BM segmentation patterns and, subsequently, to different whole-body MTV and TLG values

**Fig. 2:**
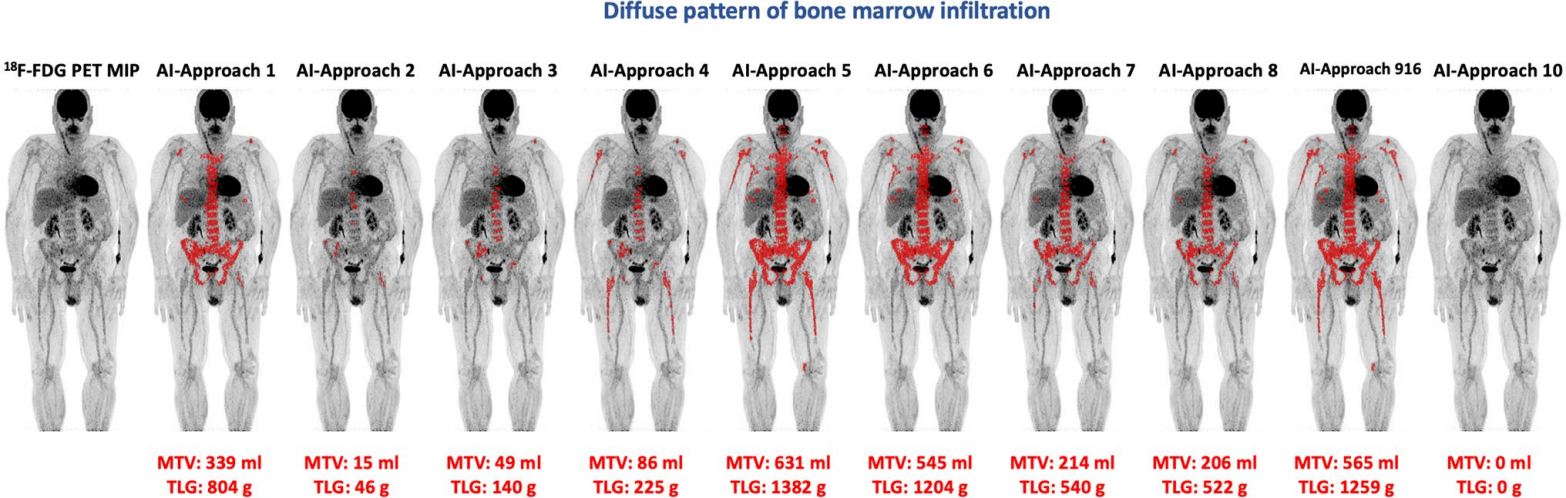
Example of the application of the AI-based software tool for automated calculation of total MTV and TLG of a MM patient with diffuse BM [^18^F]FDG uptake in the axial skeleton as well as both humeri and femora. The use of different tracer uptake thresholds leads to different BM segmentation patterns and, subsequently, to different whole-body MTV and TLG values. Notably, the application of AI approach 10 could identify no pathologically increased tracer uptake in the BM, leading to zero values for the parameters MTV and TLG

Ten different SUV thresholds were used to define pathological skeletal tracer uptake and subsequently calculate AI-based, automated whole-body MTV and TLG values. BM segmentation and volumetric calculations were feasible in all patients. The results of this analysis are shown in Table 2. Examples of the application of the tool in two patients of the studied cohort are presented in Figs. 1 and 2.

### Correlation between automated quantitative PET/CT parameters and BM plasma cell infiltration

Exploratory correlation analysis revealed a significant, moderate, positive correlation between the automated quantitative whole-body PET/CT parameters, MTV and TLG, and BM plasma cell infiltration after employment of all ten thresholds. The results of this analysis can be found in Table 2.

### Correlation between automated quantitative PET/CT parameters and patient survival

Median follow-up [95% CI] of the patient cohort from the date of PET/CT was 110 months [105–123 months]. At the time of analysis, the median PFS was 37.5 months [29.9–89.4 months] and 33 patients had progressed or died. Respectively, the median OS was not reached [104.0–NA], with 16 patients having died. Univariable analysis for both PFS and OS was performed including the automated volumetric parameters MTV and TLG derived after application of all ten thresholds. Multivariable analysis was also performed for the PET volumetric parameters, after adjusting for high-risk cytogenetic abnormalities and BM plasma cell infiltration rate.

**Fig. 3:**
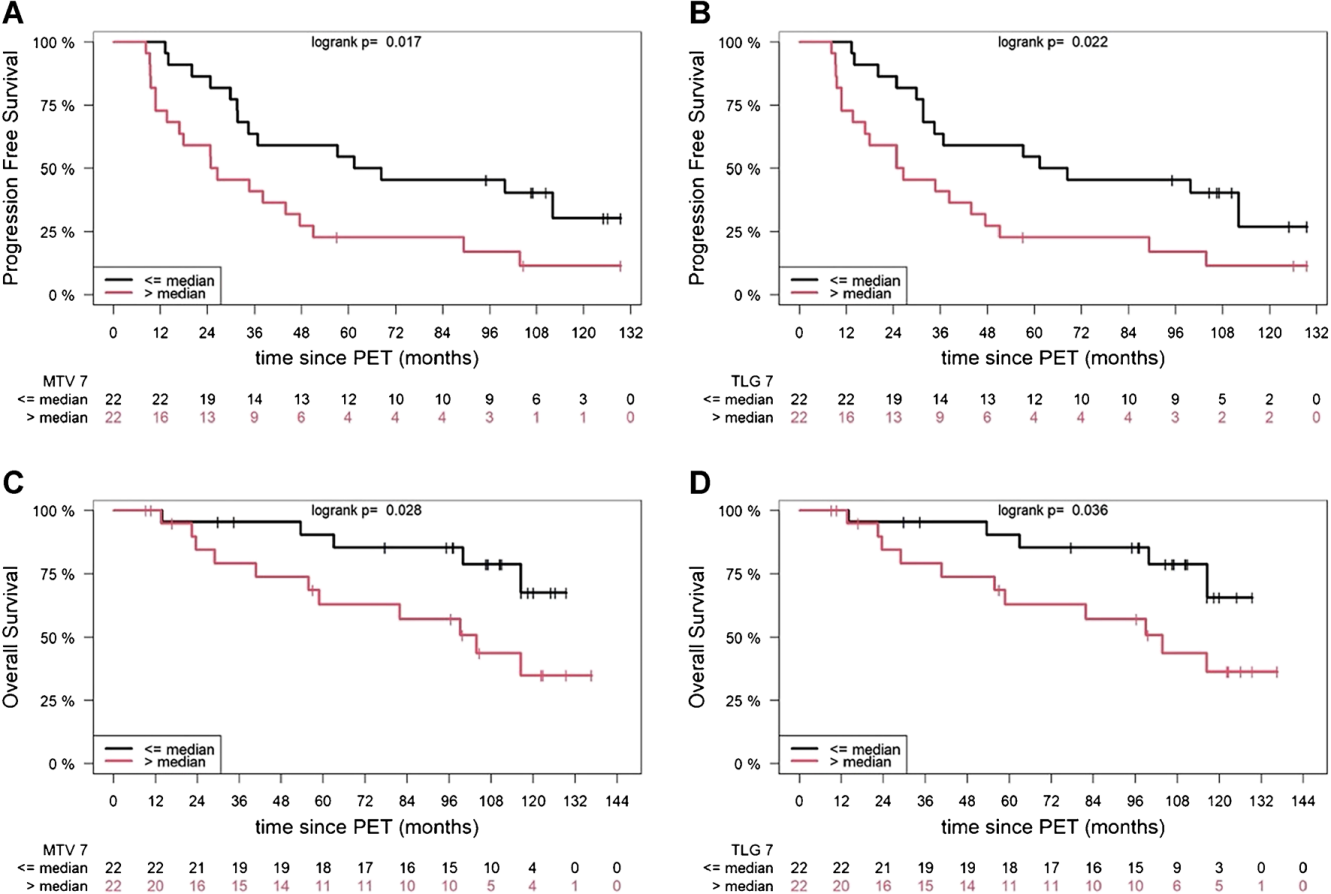
Example of Kaplan–Meier estimates of PFS according to AI-derived, whole-body MTV (**A**) and TLG (**B**), and estimates of OS according to whole-body MTV (**C**) and TLG (**D**), based on approach 7. The number of patients at risk in each group and at each time point is shown below the plots

Univariable analysis showed that both MTV and TLG predicted PFS accurately for all AI approaches (Table 3). In the multivariable analysis, high MTV based on approaches 1, 5, 6, 7, 8, and 9 retained its prognostic significance (Table 4), whereas TLG had no effect on PFS at any of the applied SUV thresholds. Further, the automated quantitative PET parameters were dichotomized at the median to investigate their effect on PFS, estimated by the Kaplan–Meier method and log-rank test. PFS curve was significantly shorter in patients with higher than the median volumetric PET values based on approaches 1, 2, 7, 8, and 10 for MTV, and approaches 1, 5, 6, 7, 8, and 9 for TLG, respectively (Fig. 3, Suppl. Figures 1–9, Suppl. Tables 1 and 2).

**Table 3:** Prognostic significance of the AI-derived PET biomarkers, according to the different SUV thresholds, for progression-free survival and overall survival in univariable analysis

		Progression-free survival		Overall survival	
AI approaches	PET parameters	HR (95% CI)	*p*-value	HR (95% CI)	*p*-value
*Approach 1*	MTV TLG	1.0012 (1.0002–1.0021) 1.0003 (1.00002–1.0005)	0.01* 0.03*	1.0014 (1.0001–1.0027) 1.0003 (0.9999–1.0007)	0.03* 0.12
*Approach 2*	MTV TLG	1.0019 (1.0002–1.0035) 1.0004 (1.0000–1.0008)	0.02* 0.04*	1.0019 (1.0000–1.0042) 1.0003 (0.9997–1.0009)	0.12 0.30
*Approach 3*	MTV TLG	1.0008 (0.9997–1.0018) 1.0002 (0.9999–1.0005)	0.14 0.15	1.0010 (1.0000–1.0025) 1.0002 (0.9998–1.0006)	0.16 0.28
*Approach 4*	MTV TLG	1.0007 (0.9997–1.0017) 1.0002 (0.9999–1.0005)	0.16 0.17	1.0010 (0.9996–1.0024) 1.0002 (0.9998–1.0006)	0.16 0.27
*Approach 5*	MTV TLG	1.0010 (1.0002–1.0018) 1.0003 (1.0000–1.0005)	0.01* 0.03*	1.0013 (1.0002–1.0024) 1.0003 (1.0000–1.0006)	0.02* 0.08
*Approach 6*	MTV TLG	1.0010 (1.0002–1.0018) 1.0003 (1.0000–1.0005)	0.01* 0.03*	1.0013 (1.0002–1.0024) 1.0003 (1.0000–1.0006)	0.02* 0.09
*Approach 7*	MTV TLG	1.0013 (1.0002–1.0024) 1.0003 (1.0000–1.0005)	0.01* 0.03*	1.0016 (1.0001–1.0030) 1.0003 (0.9999–1.0007)	0.04* 0.13
*Approach 8*	MTV TLG	1.0013 (1.0003–1.0024) 1.0003 (1.0000–1.0006)	0.01* 0.03*	1.0015 (1.0001–1.0030) 1.0003 (0.9999–1.0007)	0.04* 0.15
*Approach 9*	MTV TLG	1.0010 (1.0002–1.0018) 1.0003 (1.0000–1.0005)	0.01* 0.03*	1.0013 (1.0002–1.0024) 1.0003 (1.0000–1.0006)	0.02* 0.09
*Approach 10*	MTV TLG	1.0011 (0.9999–1.0027) 1.0002 (1.0000–1.0006)	0.19 0.19	1.0012 (0.9989–1.0034) 1.0002 (1.0000–1.0008)	0.31 0.46

*Statistically significant correlation

*HR*, hazard ratio; *95% CI*, 95% confidence intervals

**Table 4:** Multivariable model on clinical parameters and the AI-derived PET metric MTV significantly influencing progression-free survival and overall survival. The PET parameter TLG did not show any significant correlation with patient survival in the multivariable model

		Progression-free survival		Overall survival	
AI approaches	Parameters	HR (95% CI)	*p*-value	HR (95% CI)	*p*-value
*Approach 1*	MTV	1.0012 (1.0001–1.0023)	0.04*	1.0014 (0.9999–1.0029)	0.06
	BM plasma cell infiltration rate	1.0028 (0.9885–1.0172)	0.70	1.0013 (0.9813–1.0217)	0.90
	High-risk cytogenetics	1.906 (0.7058–5.1488)	0.20	1.9570 (0.4745–8.0711)	0.35
*Approach 5*	MTV	1.0010 (1.0001–1.0019)	0.04*	1.0013 (1.0001–1.0025)	0.04*
	BM plasma cell infiltration rate	1.0029 (0.9887–1.0173)	0.69	1.0008 (0.9806–1.0214)	0.94
	High-risk cytogenetics	1.8802 (0.6956–5.0820)	0.21	1.966 (0.4765–8.1109)	0.35
*Approach 6*	MTV	1.0010 (1.0001–1.0020)	0.03*	1.0013 (1.0000–1.0026)	0.04*
	BM plasma cell infiltration rate	1.0027 (0.9885–1.0172)	0.71	1.0009 (0.9808–1.0215)	0.93
	High-risk cytogenetics	1.9358 (0.7155–5.2375)	0.19	2.0208 (0.4875–8.3773)	0.33
*Approach 7*	MTV	1.0013 (1.0001–1.0026)	0.04*	1.0015 (0.9998–1.0032)	0.08
	BM plasma cell infiltration rate	1.0030 (0.9888–1.0174)	0.68	1.0017 (0.9818–1.0220)	0.87
	High-risk cytogenetics	1.8851 (0.6986–5.0871)	0.21	1.9151 (0.4656–7.8765)	0.37
*Approach 8*	MTV	1.0013 (1.0001–1.0026)	0.04*	1.0015 (1.0000–1.0032)	0.09
	BM plasma cell infiltration rate	1.0030 (0.9887–1.0174)	0.68	1.0017 (0.9818–1.0220)	0.87
	High-risk cytogenetics	1.8958 (0.7027–5.1149)	0.21	1.9125 (0.4653–7.8606)	0.37
*Approach 9*	MTV	1.0010 (1.0001–1.0019)	0.04*	1.0013 (1.0000–1.0026)	0.04*
	BM plasma cell infiltration rate	1.0028 (0.9885–1.0173)	0.70	1.0007 (0.9805–1.0214)	0.94
	High-risk cytogenetics	1.8846 (0.6976–5.0909)	0.21	1.9704 (0.4771–8.1384)	0.35

*Statistically significant correlation

Multivariable analyses were carried out using a Cox regression model

*HR*, hazard ratio; *95% CI*, 95% confidence intervals; *BM*, bone marrow; *MTV*, metabolic tumor volume; *TLG*, total lesion glycolysis

Univariable analysis for OS showed that whole-body MTV based on approaches 1, 5, 6, 7, 8, and 9 was significantly associated with shorter survival (Table 3), whereas TLG had no effect on OS. Accordingly, in the multivariable regression model for OS, MTV derived from approaches 5, 6, and 9 was significantly associated with shorter OS, while a similar though non-significant trend was also observed for MTV based on approaches 1, 7, and 8 (Table 4). No significant results were obtained with high TLG values in multivariable analysis with any of the SUV thresholds used. After dichotomization of the PET parameters at the median, the log-rank test showed that the OS curve was significantly shorter in patients with automated PET values higher than the median based on approaches 1, 7, and 8 for MTV, and approaches 1, 5, 6, 7, 8, and 9 for TLG, respectively (Fig. 3, Suppl. Figures 1–9, Suppl. Tables 1 and 2).

**Fig. 4:**
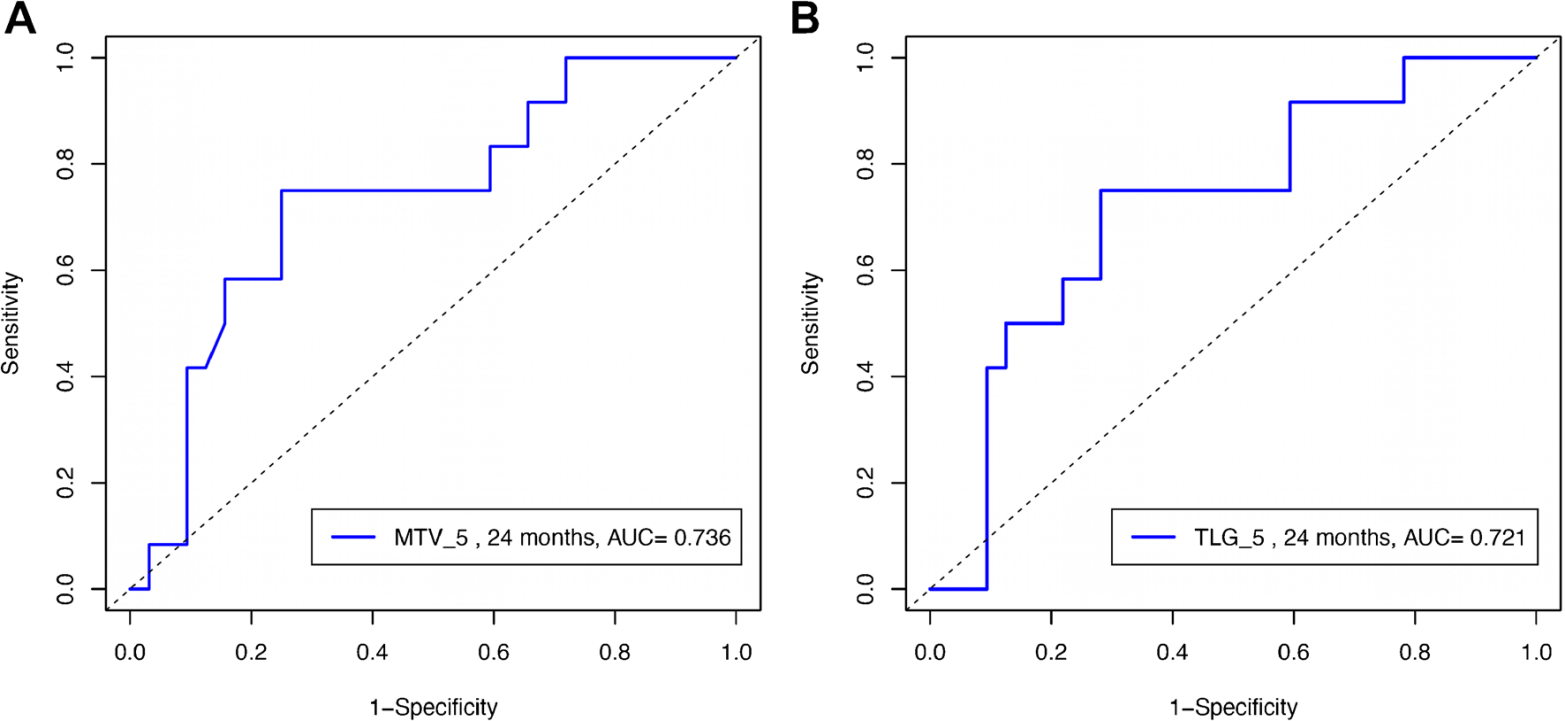
ROC curves for prediction of 2-year PFS from the time of PET/CT imaging based on approach 5. The cut points of absolute volumetric PET values for optimizing the sum of sensitivity and specificity were 394.0 mL for MTV (sensitivity = 0.75, specificity = 0.75; **A**) and 1015.1 g for TLG (sensitivity = 0.75, specificity = 0.72; **B**)

Further, in an attempt to evaluate the discrimination performance of the automated tool in correctly predicting PFS over a time interval of 2 years, we performed ROC analysis for all approaches. In line with the previous, liver-based cut-offs, in particular approaches 1, 5, 6, 7, 8, and 9 provided the best results for PFS prediction, as reflected by the respective AUC being significantly different from 0.5 for both MTV and TLG. Cut points of absolute values of the MTV and TLG parameters for optimizing the sum of sensitivity and specificity were identified. Due to the small number of events (*n* = 4), we did not perform ROC-based calculations for 2-year OS. The detailed results of ROC analysis are presented in Table 5. An example of ROC curves is presented in Fig. 4.

**Table 5:** Area under the curve (AUC), 95% confidence interval (95% CI) of AUC, *p*-values for testing whether AUC = 0.5, and MTV and TLG thresholds optimizing the sum of sensitivity and specificity at this threshold according to the different approaches for prediction of 2-year PFS from the time of PET/CT imaging

AI approaches	PET parameters	Thresholds	AUC	95% CI	*p*	Sensitivity	Specificity
Approach 1	MTV (mL)	363.5	0.723	0.549–0.896	0.01*	0.58	0.88
	TLG (g)	707.6	0.727	0.556–0.897	< 0.01*	0.75	0.72
Approach 2	MTV (mL)	107.5	0.702	0.510–0.893	0.04*	0.58	0.88
	TLG (g)	129.0	0.688	0.494–0.881	0.06	0.75	0.69
Approach 3	MTV (mL)	188.5	0.682	0.497–0.868	0.05	0.75	0.75
	TLG (g)	520.6	0.690	0.511–0.869	0.04*	0.75	0.72
Approach 4	MTV (mL)	195.0	0.671	0.485–0.856	0.07	0.75	0.72
	TLG (g)	587.1	0.685	0.504–0.866	0.04*	0.75	0.72
Approach 5	MTV (mL)	394.0	0.736	0.567–0.905	< 0.01*	0.75	0.75
	TLG (g)	1015.1	0.721	0.551–0.892	0.01*	0.75	0.72
Approach 6	MTV (mL)	384.5	0.724	0.555–0.893	< 0.01*	0.75	0.72
	TLG (g)	1002.9	0.724	0.553–0.894	0.01*	0.75	0.72
Approach 7	MTV (mL)	265	0.734	0.564–0.904	< 0.01*	0.88	0.58
	TLG (g)	811.1	0.729	0.559–0.899	< 0.01*	0.88	0.58
Approach 8	MTV (mL)	267.0	0.729	0.560–0.898	< 0.01*	0.88	0.58
	TLG (g)	811.1	0.729	0.561–0.898	< 0.01*	0.84	0.58
Approach 9	MTV (mL)	428.0	0.736	0.565–0.906	< 0.01*	0.67	0.81
	TLG (g)	1174.8	0.737	0.567–0.907	< 0.01*	0.67	0.81
Approach 10	MTV (mL)	49.5	0.669	0.479–0.859	0.08	0.75	0.72
	TLG (g)	194.5	0.674	0.483–0.866	0.07	0.75	0.72

*Statistically significant results

*AUC*, area under the curve; *95% CI*, 95% confidence interval; *MTV*, metabolic tumor volume

### Application of IMPeTUs

The use of IMPeTUs yielded the following results: the median 5-point DS of diffuse BM uptake was 3 (range DS = 2–5). Eleven patients (25%) had no detectable focal medullary hypermetabolic lesions (F1 score), while 33 of them (75%) had at least one focal hypermetabolic lesion (median F score = 2; range F score = 1–4). In the 33 patients with focal lesions, the median 5-point DS of the hottest lesion was 5 (range DS = 3–5). PMD and EMD were present in 22/44 (50%) and 4/44 (9%) patients, respectively. Eight patients (18%) had no osteolysis (L1 score), while 36 of them (82%) had at least one lytic lesion (median L score = 3; range L score = 1–4). Fractures were found in 20 patients (45%) (Suppl. Table 3).

Univariable analysis revealed that the number of focal, [^18^F]FDG-avid lesions and the presence of EMD were significantly associated with shorter PFS, whereas the other parameters considered in IMPeTUs had no effect on PFS. No parameter had a significant effect on OS, although a non-significant (*p* = 0.06) trend towards shorter OS was observed in patients with more hypermetabolic lesions. The results of this analysis are shown in Suppl. Table 4.

Similar to the automated PET volumetric parameters, multivariable analysis was performed for the IMPeTUs parameters after adjustment for high-risk cytogenetic abnormalities and BM plasma cell infiltration rate. The number of focal, [^18^F]FDG-avid, BM lesions and the presence of EMD were associated with significantly shorter PFS, while the other parameters did not have a significant effect on PFS. No parameter had a significant effect on OS.

## Discussion

MM represents a heterogeneous hematological malignancy with a highly variable clinical outcome [2]. Therefore, the identification of reliable prognostic factors and robust positivity cut-offs for outcome prediction have beneficial implications for disease management. In this context, the volumetric PET indices MTV and TLG have emerged in recent years as promising metabolic parameters for the quantification of tumor burden and patient prognosis in MM, although no standardized methodology for their calculation has yet been established [13, 14, 23]. Accordingly, the aim of this study was to investigate the prognostic role of automated, baseline, volumetric PET/CT calculations of BM metabolism in patients with newly diagnosed MM, using an AI-based tool that has recently been shown to be satisfactorily applicable to the assessment of myeloma disease burden [16].

The major findings of our study are the following: firstly, we confirm the significant positive correlation between the AI-derived, whole-body parameters MTV and TLG and the degree of BM plasma cell infiltration, initially demonstrated in our previous study [16]. Secondly, based on univariable analysis, the MTV and TLG values are significantly associated with adverse patient outcome after application of several different [^18^F]FDG uptake thresholds for automated BM segmentation. Lastly, using liver [^18^F]FDG uptake, partly with some modifications, as a cut-off to define patient-level pathological tracer uptake in the BM, whole-body MTV is associated with poor PFS and OS in multivariable analysis adjusting for high-risk cytogenetic abnormalities and BM plasma cell infiltration rate.

PET-based volumetric calculation of total tumor burden has gained increasing interest as a more reliable way of estimating disease extent and therefore prognosis [24–26]. Nevertheless, this approach has not yet become established in daily nuclear medicine practice for clinical interpretation because its application requires accurate identification and segmentation of tumor lesions, as well as a degree of manual correction of segmentation and exclusion of tracer uptake in normal organs or other non-malignant sites [27, 28]. Particularly in MM, this process of exact tumor delineation can be cumbersome and time-consuming as these patients often have multiple lesions and different patterns of BM involvement, with both focal and diffuse bone lesions coexisting with varying degrees of [^18^F]FDG uptake. It would not be an exaggeration to claim that in some MM patients, accurate quantification of tumor volume is virtually impossible and is hampered by subjective evaluation due to the extensive infiltration of the BM and its variable patterns. Recognizing this difficulty and the lack of consensus on the best method for PET measurements in MM, we have recently applied an AI-based method with the aim of standardizing tumor volume quantification at the whole-body level. Having demonstrated the feasibility of the tool for automated volumetric quantification of BM infiltration, and the significant correlation of the therein generated whole-body MTV and TLG parameters with the results of conventional analysis of PET/CT findings [16], in the present work, we were able to confirm the significant positive correlation of these parameters with the degree of BM infiltration by plasma cells after application of ten different uptake thresholds in another MM cohort. Despite the sampling error in BM biopsies due to the spatial heterogeneity of MM in the BM compartment, this finding underlines the reproducibility and robustness of the AI tool for volumetric calculations, while also highlighting the suitability of these PET parameters for the assessment of histopathological tumor burden in MM, as suggested by previous studies [23, 29].

Nevertheless, the main aim of our work was to investigate the prognostic role of the AI-derived volumetric parameters in relation to outcome in patients with MM. Here, the results did indeed demonstrate the potential of automated quantification of total metabolic load as a tool for predicting survival. Starting with univariable analysis, the parameters MTV and TLG generated by most segmentation approaches were found to be predictive of PFS. Specifically, seven of the ten thresholds used (approaches 1, 2, 5, 6, 7, 8, and 9) showed that high MTV and TLG had a significant adverse effect on PFS. Furthermore, on multivariable analysis, MTV was found to be an independent predictor of PFS after applying six of the above SUV thresholds (approaches 1, 5, 6, 7, 8, and 9). Accordingly, in terms of OS, MTV had a significant effect on patient outcome both on univariable (approaches 1, 5, 6, 7, 8, and 9) and multivariable analysis (approaches 5, 6, and 9). A common feature of the approaches with a significant impact on survival is that, with some modifications, they use liver uptake as a threshold to define pathological [^18^F]FDG uptake in the BM, a finding also confirmed by the results of Kaplan–Meier analysis. These results support the role of patient-level segmentation using the liver as background reference organ in the evaluation of myeloma with PET/CT [10, 11, 15]. Further, given these findings, a robust yet simple and practical threshold to predict patient survival in MM would be the mean or median value of liver [^18^F]FDG uptake which could reliably serve as a reference for automatic BM segmentation.

In the same context, although some cut-off values for whole-body MTV and TLG for predicting 2-year PFS have been identified using ROC analysis, we prefer not to recommend these absolute values as general thresholds for predicting survival in MM. In contrast to other studies in this field, which relied exclusively on absolute tracer uptake values to identify abnormal glucose metabolism in the BM compartment and subsequently led to the generation of corresponding volumetric thresholds [12, 14, 23], in our work, thresholds based on liver uptake per patient performed better than absolute SUV cut-off values. On the basis of these findings, we therefore advocate the use of patient-level liver-based segmentation thresholds, in line with previous studies that have highlighted BM metabolism relative to liver uptake as a useful and reproducible approach to PET image interpretation and as a reliable predictor of outcome, especially in MM patients receiving ASCT [10, 30]. Interestingly, the use of liver [^18^F]FDG uptake as a threshold to classify BM uptake within the IMPeTUs criteria did not have a significant effect on survival, which may be due to the relatively small patient cohort. On the other hand, the number of focal hypermetabolic lesions and EMD had a significant adverse effect on PFS, which is consistent with previous analysis by our group in this cohort [20].

The identification of MTV as a negative predictor in MM is consistent with previous works [12–14, 23]. However, the main novelty of the present study is the use of an automated, quick, and simple global thresholding tool for bone segmentation and subsequent quantification of [^18^F]FDG metabolism, which is not affected by manual or semi-automated region of interest (ROI) definitions, and thus operator-independent. In particular, the deep learning, volumetric quantification method applied here is based on the initial CT-based segmentation of the skeleton, its transfer to the PET images and the application of different tracer uptake thresholds, and the subsequent refinement of the resulting regions using postprocessing. In fact, this is an area where AI can find broad application, as it can surpass older, conventional methods of whole-body volumetric analysis and can be used as an adjunct and very facilitating tool for interpreting physicians, removing a significant amount of repetitive, time-consuming, operator-dependent, and tedious tasks [28]. An additional strength of our analysis lies in the follow-up period of the cohort, which is, to our knowledge, the longest published in the field of PET/CT studies focusing on application of whole-body, volumetric metabolic parameters in MM patients.

This study has certain limitations. First, this is a single-center retrospective analysis of prospectively acquired data from a relatively small cohort. Ideally, data from larger, prospective, multicentric studies would be warranted to validate the findings presented here. However, the cohort studied is homogeneous, consisting of treatment-naïve MM patients at the time of PET/CT, who have received very similar therapies and have been followed for a very long time. Second, there are limitations in the segmentation method used: the calculation of MTV and TLG is SUV-dependent, meaning that the calculation of these parameters is susceptible to a number of factors that affect SUV, such as partial volume correction, blood glucose, reconstruction and acquisition parameters, and time between [^18^F]FDG injection and image acquisition [16]. Further weaknesses of the applied methodology include the exclusion of the skull from calculations due to the very high [^18^F]FDG uptake in the brain, which requires an independent assessment of this anatomical area, inevitably making the method more operator-dependent in selected MM cases with cranial involvement. Another source of error that may potentially require manual corrections is extensive lytic or paramedullary lesions, which may be excluded from the BM segmentation, as the AI tool is based on the initial CT-based identification of the skeleton using the Hounsfield unit (HU) scale of each region. These specific lesions will be investigated in a larger patient cohort in terms of another multi-center, randomized phase 3 trial [31] with the aim of validating AI-based volumetric PET calculations against whole-body MRI, which is considered the modality of choice for BM assessment in MM patients [7]. Finally, although the patients studied received novel agents at that time, the therapeutic landscape in MM is rapidly changing, with newer agents such as monoclonal antibodies, bispecific antibodies, and CAR-T cells being approved or tested for the treatment of the disease. It would therefore be interesting to validate the prognostic performance of the tool in cohorts receiving such therapies.

## Conclusion

In this study, we investigated the prognostic role of automatically obtained whole-body volumetric calculations of BM metabolism after application of an automated, deep learning–based tool on PET/CT images in a cohort of 44 MM patients. At a median follow-up of 110 months [105–123 months], based on univariable analysis, the AI-derived parameters MTV and TLG had a significant adverse effect on patient outcome after application of several different [^18^F]FDG uptake thresholds for automated BM segmentation. Importantly, in multivariable analysis adjusted for high-risk cytogenetic abnormalities and BM plasma cell infiltration rate, whole-body MTV was significantly associated with poor PFS and OS. In addition to highlighting the prognostic significance of automated, global volumetric calculations of metabolic tumor burden, using the liver uptake as reference for BM segmentation, these data open up new perspectives towards solving the complex problem of interpreting PET scans in MM patients with a simple, fast, and robust method that is not affected by manual or semi-automated, and thus operator-dependent, interventions.

## Supplementary Information

Below is the link to the electronic supplementary material.

Supplementary file1 (DOCX 3.47 KB)

## Data Availability

The datasets generated and/or analyzed during the current study are available from the corresponding author on reasonable request.
