# Correction to: Artificial intelligence–based, volumetric assessment of the bone marrow metabolic activity in [^18^F]FDG PET/CT predicts survival in multiple myeloma

**DOI:** 10.1007/s00259-025-07158-6

**Published:** 2025-02-22

**Authors:** Christos Sachpekidis, Olof Enqvist, Johannes Ulén, Annette Kopp‑Schneider, Leyun Pan, Elias K. Mai, Marina Hajiyianni, Maximilian Merz, Marc S. Raab, Anna Jauch, Hartmut Goldschmidt, Lars Edenbrandt, Antonia Dimitrakopoulou‑Strauss

**Affiliations:** 1 Clinical Cooperation Unit Nuclear Medicine, German Cancer Research Center (DKFZ), Im Neuenheimer Feld 280, 69210 Heidelberg, Germany; 2 Eigenvision AB, Malmö, Sweden; 3 Department of Electrical Engineering, Chalmers University of Technology, Gothenburg, Sweden; 4 Division of Biostatistics, German Cancer Research Center (DKFZ), Heidelberg, Germany; 5 Department of Internal Medicine V, University Hospital Heidelberg and National Center for Tumor Diseases (NCT), Heidelberg, Germany; 6 Department of Hematology and Cell Therapy, University of Leipzig, Leipzig, Germany; 7 Institute of Human Genetics, University of Heidelberg, Heidelberg, Germany; 8 Department of Clinical Physiology, Region Västra Götaland, Sahlgrenska University Hospital, Gothenburg, Sweden; 9 Department of Molecular and Clinical Medicine, Institute of Medicine, Sahlgrenska Academy, University of Gothenburg, Gothenburg, Sweden

**Correction to: European Journal of Nuclear Medicine and Molecular Imaging (2024) 51:2293–2307**

10.1007/s00259-024-06668-z

The authors regret that the version of Fig. 3 that appears in the original published article is incorrect.

Below is the incorrect Fig. 3.

**Fig. 3:**
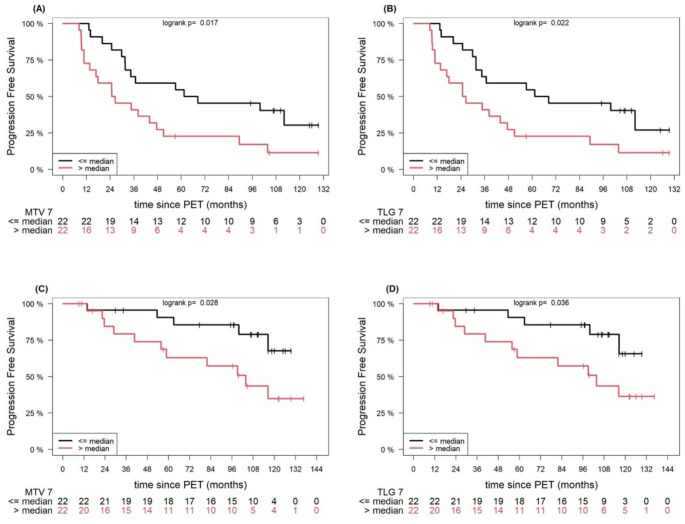
Example of Kaplan-Meier estimates of PFS according to AI-derived, whole-body MTV (**A**) and TLG (**B**), and estimates of OS according to whole-body MTV (**C**) and TLG (**D**), based on approach 7. The number of patients at risk in each group and at each time point is shown below the plots

The correct Fig. 3 is shown below.

**Fig. 3:**
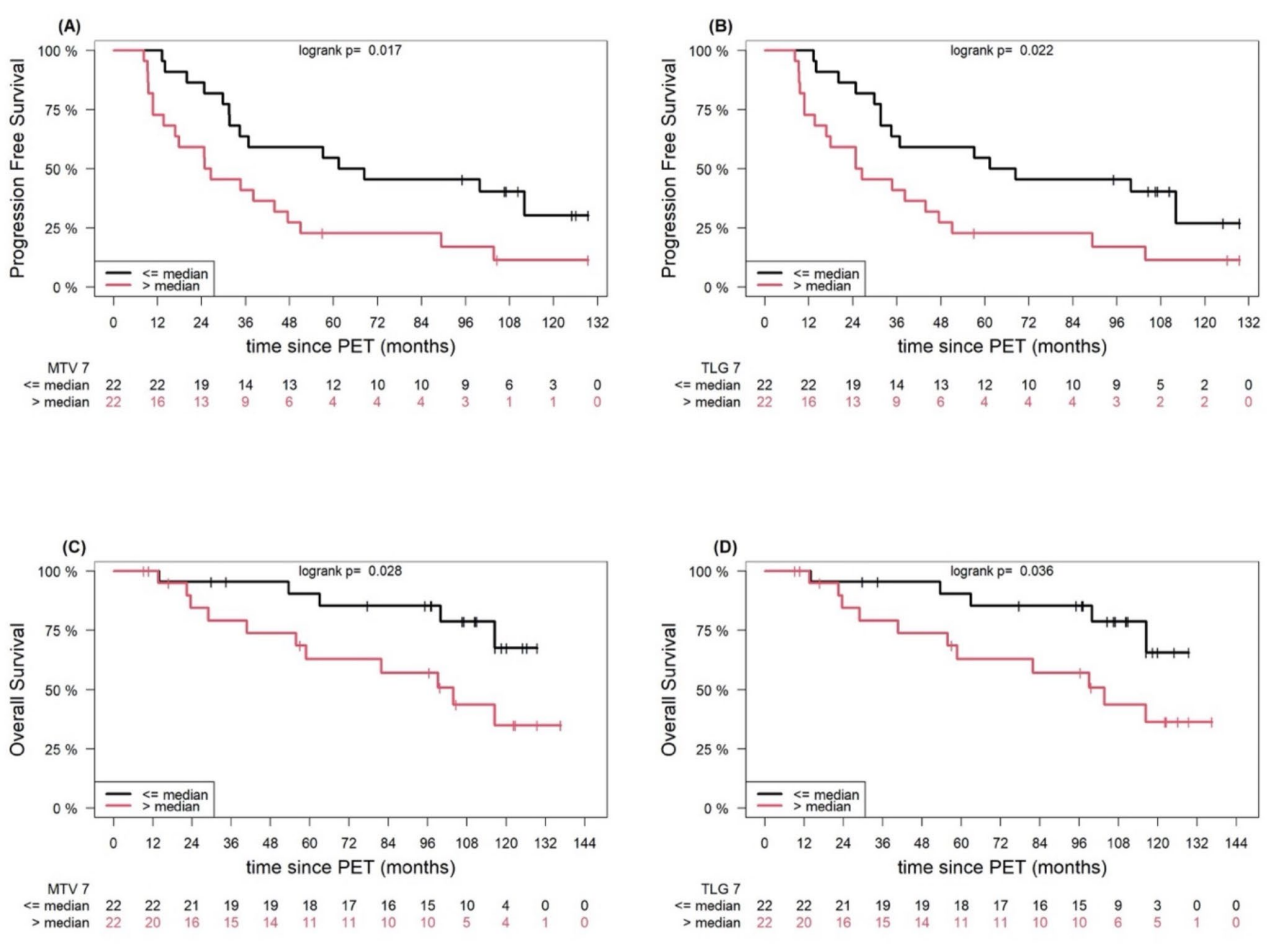
Example of Kaplan-Meier estimates of PFS according to AI-derived, whole-body MTV (**A**) and TLG (**B**), and estimates of OS according to whole-body MTV (**C**) and TLG (**D**), based on approach 7. The number of patients at risk in each group and at each time point is shown below the plots

The original article has been corrected.

