# Supplementary material for: Artificial intelligence–based, volumetric assessment of the bone marrow metabolic activity in [18F]FDG PET/CT predicts survival in multiple myeloma
Source: Eur J Nucl Med Mol Imaging. 2024 Mar 8;51(8):2293–307. doi: 10.1007/s00259-024-06668-z (PMC11178614; doi:10.1007/s00259-024-06668-z)

**Supplementary Table 1** Results of survival (Kaplan-Meier) analysis based on AI-derived whole-body MTV values according to the different SUV thresholds.

| **Parameter** |  | **Baseline PET/CT** | | | |
| --- | --- | --- | --- | --- | --- |
|  |  | **Median PFS [95% CI]** | ***p value*** | **Median OS [95% CI]** | ***p value*** |
| **MTV 1** | ≤ median | 64.9 months [34.5 – NA] | 0.02* | NA [116.4 – NA] | 0.03* |
|  | > median | 25.7 months [16.8 – 51.1] |  | 104.0 months [58.8 – NA] |  |
| **MTV 2** | ≤ median | 68.4 months [34.5 – NA] | 0.02* | NA [116.4 – NA] | 0.23 |
|  | > median | 25.7 months [13.7 – 51.1] |  | 116.0 months [58.8 – NA] |  |
| **MTV 3** | ≤ median | 59.3 months [31.6 – NA] | 0.13 | NA [116.4 – NA] | 0.18 |
|  | > median | 30.6 months [16.8 – 89.4] |  | 99.9 months [81.8 – NA] |  |
| **MTV 4** | ≤ median | 59.3 months [31.6 – NA] | 0.13 | NA [116.4 – NA] | 0.18 |
|  | > median | 30.6 months [16.8 – 89.4] |  | 99.9 months [81.8 – NA] |  |
| **MTV 5** | ≤ median | 59.3 months [31.6 – NA] | 0.09 | NA [116.4 – NA] | 0.12 |
|  | > median | 29.8 months [16.8 – 89.4] |  | 104.0 months [81.8 – NA] |  |
| **MTV 6** | ≤ median | 59.3 months [31.6 – NA] | 0.09 | NA [116.4 – NA] | 0.12 |
|  | > median | 29.8 months [16.8 – 89.4] |  | 104.0 months [81.8 – NA] |  |
| **MTV 7** | ≤ median | 64.9 months [34.5 – NA] | 0.02* | NA [116.4 – NA] | 0.03* |
|  | > median | 25.7 months [16.8 – 51.1] |  | 104.0 months [58.8 – NA] |  |
| **MTV 8** | ≤ median | 64.9 months [34.5 – NA] | 0.02* | NA [116.4 – NA] | 0.03* |
|  | > median | 25.7 months [16.8 – 51.1] |  | 104.0 months [58.8 – NA] |  |
| **MTV 9** | ≤ median | 59.3 months [34.5 – NA] | 0.07 | NA [116.4 – NA] | 0.12 |
|  | > median | 25.7 months [16.8 – 89.4] |  | 104.0 months [81.8 – NA] |  |
| **MTV 10** | ≤ median | 64.9 months [34.5 – NA] | 0.02* | NA [116.4 – NA] | 0.12 |
|  | > median | 29.0 months [16.8 – 51.1] |  | 99.9 months [81.8 – NA] |  |

* Statistically significant difference

SUV, standardized uptake value; MTV, metabolic tumor volume; PFS, progression-free survival; OS, overall survival; NA, not applicable.

**Supplementary Table 2** Results of survival (Kaplan-Meier) analysis based on AI-derived whole-body TLG values according to the different SUV thresholds.

| **Parameter** |  | **Baseline PET/CT** | | | |
| --- | --- | --- | --- | --- | --- |
|  |  | **Median PFS [95% CI]** | ***p value*** | **Median OS [95% CI]** | ***p value*** |
| **TLG 1** | ≤ median | 64.9 months [34.5 – NA] | 0.02* | NA [116.4 – NA] | 0.03* |
|  | > median | 25.7 months [16.8 – 51.1] |  | 104.0 months [58.8 – NA] |  |
| **TLG 2** | ≤ median | 57.1 months [31.6 – NA] | 0.12 | NA [116.4 – NA] | 0.120 |
|  | > median | 30.6 months [13.7 – 61.4] |  | 116.0 months [58.8 – NA] |  |
| **TLG 3** | ≤ median | 59.3 months [31.6 – NA] | 0.13 | NA [116.4 – NA] | 0.18 |
|  | > median | 30.6 months [16.8 – 89.4] |  | 99.9 months [81.8 – NA] |  |
| **TLG 4** | ≤ median | 59.3 months [31.6 – NA] | 0.13 | NA [116.4 – NA] | 0.18 |
|  | > median | 30.6 months [16.8 – 89.4] |  | 99.9 months [81.8 – NA] |  |
| **TLG 5** | ≤ median | 64.9 months [34.5 – NA] | 0.02* | NA [116.4 – NA] | 0.03* |
|  | > median | 25.7 months [16.8 – 51.1] |  | 99.9 months [58.8 – NA] |  |
| **TLG 6** | ≤ median | 64.9 months [34.5 – NA] | <0.01* | NA [NA – NA] | <0.01* |
|  | > median | 25.7 months [16.8 – 51.1] |  | 99.9 months [58.8 – NA] |  |
| **TLG 7** | ≤ median | 64.9 months [34.5 – NA] | 0.02* | NA [116.4 – NA] | 0.04* |
|  | > median | 25.7 months [16.8 – 51.1] |  | 104.0 months [58.8 – NA] |  |
| **TLG 8** | ≤ median | 64.9 months [34.5 – NA] | 0.02* | NA [116.4 – NA] | 0.04* |
|  | > median | 25.7 months [16.8 – 51.1] |  | 104.0 months [58.8 – NA] |  |
| **TLG 9** | ≤ median | 64.9 months [34.5 – NA] | 0.02* | NA [116.4 – NA] | 0.03* |
|  | > median | 25.7 months [16.8 – 51.1] |  | 104.0 months [58.8 – NA] |  |
| **TLG 10** | ≤ median | 59.3 months [31.6 – NA] | 0.109 | NA [116.4 – NA] | 0.15 |
|  | > median | 30.6 months [16.8 – 89.4] |  | 99.9 months [81.8 – NA] |  |

* Statistically significant difference

SUV, standardized uptake value; TLG, total lesion glycolysis; PFS, progression-free survival; OS, overall survival; NA, not applicable.

**Supplementary Table 3** PET/CT results after application of IMPeTUs.

| **IMPeTUs criteria** | Patients (%)^§^ |
| --- | --- |
| *Bone marrow uptake, DS* |  |
| 1 | 0 |
| 2 | 6 (14%) |
| 3 | 18 (41%) |
| 4 | 17 (39%) |
| 5 | 3 (7%) |
| *No. of focal, hypermetabolic lesions* |  |
| F_1_ (none) | 11 (25%) |
| F_2_ (1 - 3) | 12 (27%) |
| F_3_ (4 - 10) | 9 (20%) |
| F_4_ (> 10) | 12 (27%) |
| *Site of focal lesions** |  |
| Skull | 2 (5%) |
| Spine | 22 (50%) |
| Other | 30 (68%) |
| *Uptake of the hottest focal lesion, DS^#^* |  |
| 1 | 0 |
| 2 | 0 |
| 3 | 1 (3%) |
| 4 | 12 (36%) |
| 5 | 20 (61%) |
| *No. of lytic lesions* |  |
| L_1_ (none) | 8 (18%) |
| L_2_ (1 - 3) | 11 (25%) |
| L_3_ (4 - 10) | 5 (11%) |
| L_4_ (> 10) | 20 (45%) |
| *Presence of at least one fracture* |  |
| no | 24 (55%) |
| yes | 20 (45%) |
| *Presence of PMD** |  |
| no | 22 (50%) |
| yes | 22 (50%) |
| *Presence of EMD** |  |
| no | 40 (91%) |
| yes | 4 (9%) |

^§^ Due to rounding, the % percentage values do not necessarily add to 100%.

***** Measurements refer to hypermetabolic lesions.

**^#^**  Measurements refer to the 33 patients with detectable focal medullary hypermetabolic lesions.

**Supplementary Table 4** Predictors of progression-free survival and overall survival by univariable analysis based on PET/CT results after application of IMPeTUs.

| **IMPeTUs criteria** | **Progression-free survival** | | **Overall survival** | |
| --- | --- | --- | --- | --- |
|  | **HR (95% CI)** | **p-value** | **HR (95% CI)** | **p-value** |
| *Bone marrow uptake, DS score^#^* | 1.4326 (0.7211 - 2.8463) | 0.30 | 1.6113 (0.5972 - 4.3474) | 0.35 |
| *No. of focal, hypermetabolic lesions* | 1.6208 (1.1766 - 2.2327) | <0.01 | 1.5319 (0.9871 - 2.3771) | 0.06 |
| *Uptake of the hottest focal lesion, DS*^§^ | NA | - | NA | - |
| *No. of lytic lesions* | 1.1049 (0.8309 - 1.4693) | 0.49 | 0.8455 (0.5537 - 1.2911) | 0.44 |
| *Presence of at least one fracture* | 1.1794 (0.5921 - 2.3492) | 0.64 | 2.0612 (0.7425 - 5.7219) | 0.17 |
| *Presence of PMD* | 1.6199 (0.8030 - 3.2678) | 0.18 | 1.0390 (0.3892 - 2.7736) | 0.94 |
| *Presence of EMD* | 3.3750 (1.1495 - 9.9087) | 0.03 | 1.4499 (0.3284 - 6.4011) | 0.62 |

* Statistically significant correlation

^#^ Based on the dichotomisation of the parameter in DS< 4 and DS ≥4.

^§^ Not applicable because the number of patients with DS< 4 was very small (n= 1 patient).

DS, Deauville score; HR, hazard ratio; 95% CI, 95% confidence intervals; NA, not applicable; PMD, paramedullary

disease; EMD, extramedullary disease.

**Supplementary Figure 1** Kaplan–Meier estimates of PFS according to AI-derived, whole-body MTV (A) and TLG (B) as well as estimates of OS according to whole-body MTV (C) and TLG (D), based on approach 1. The numbers of patients at risk in each group and for the respective time points are shown below the plots.


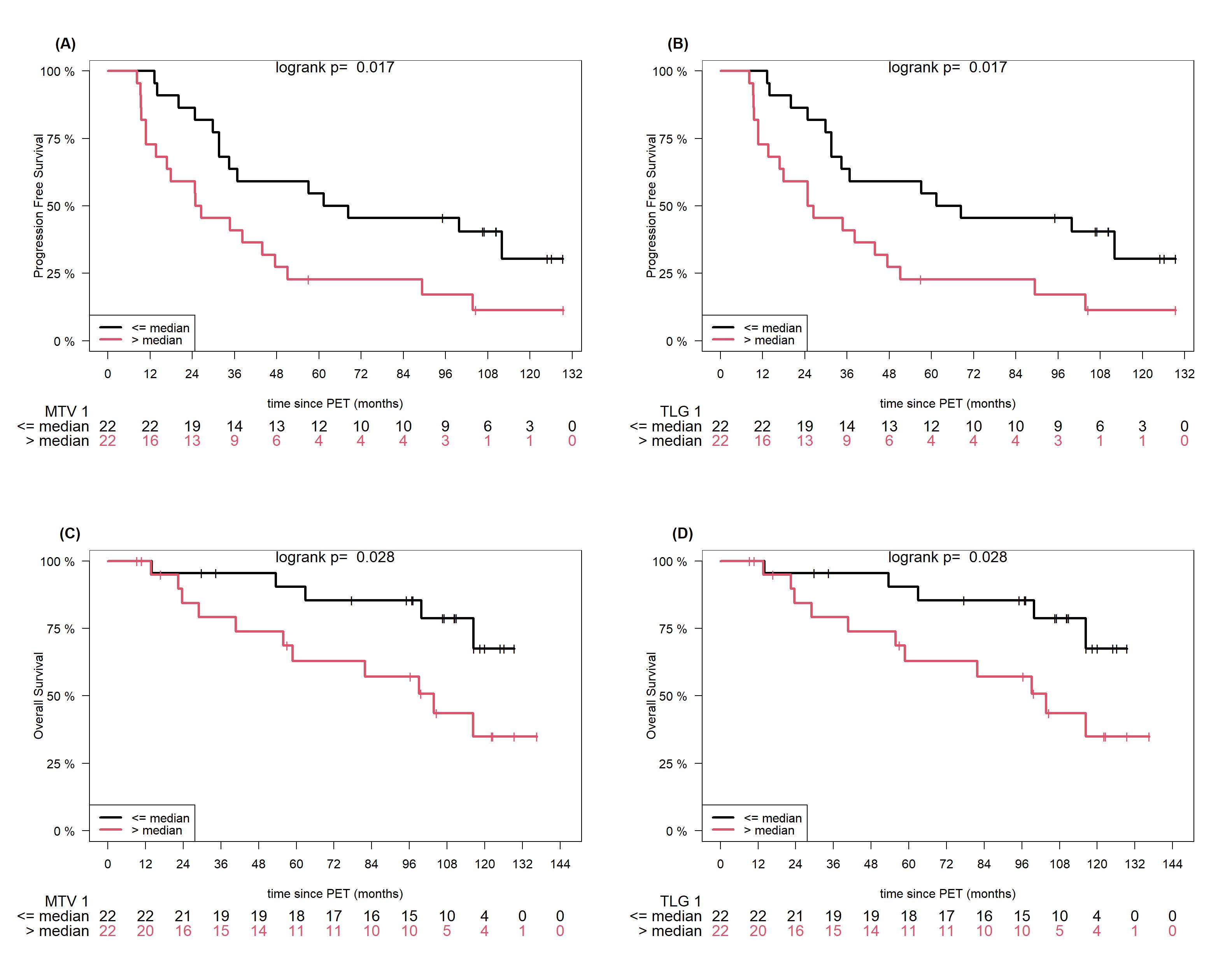


**Supplementary Figure 2** Kaplan–Meier estimates of PFS according to AI-derived, whole-body MTV (A) and TLG (B) as well as estimates of OS according to whole-body MTV (C) and TLG (D), based on approach 2. The numbers of patients at risk in each group and for the respective time points are shown below the plots.


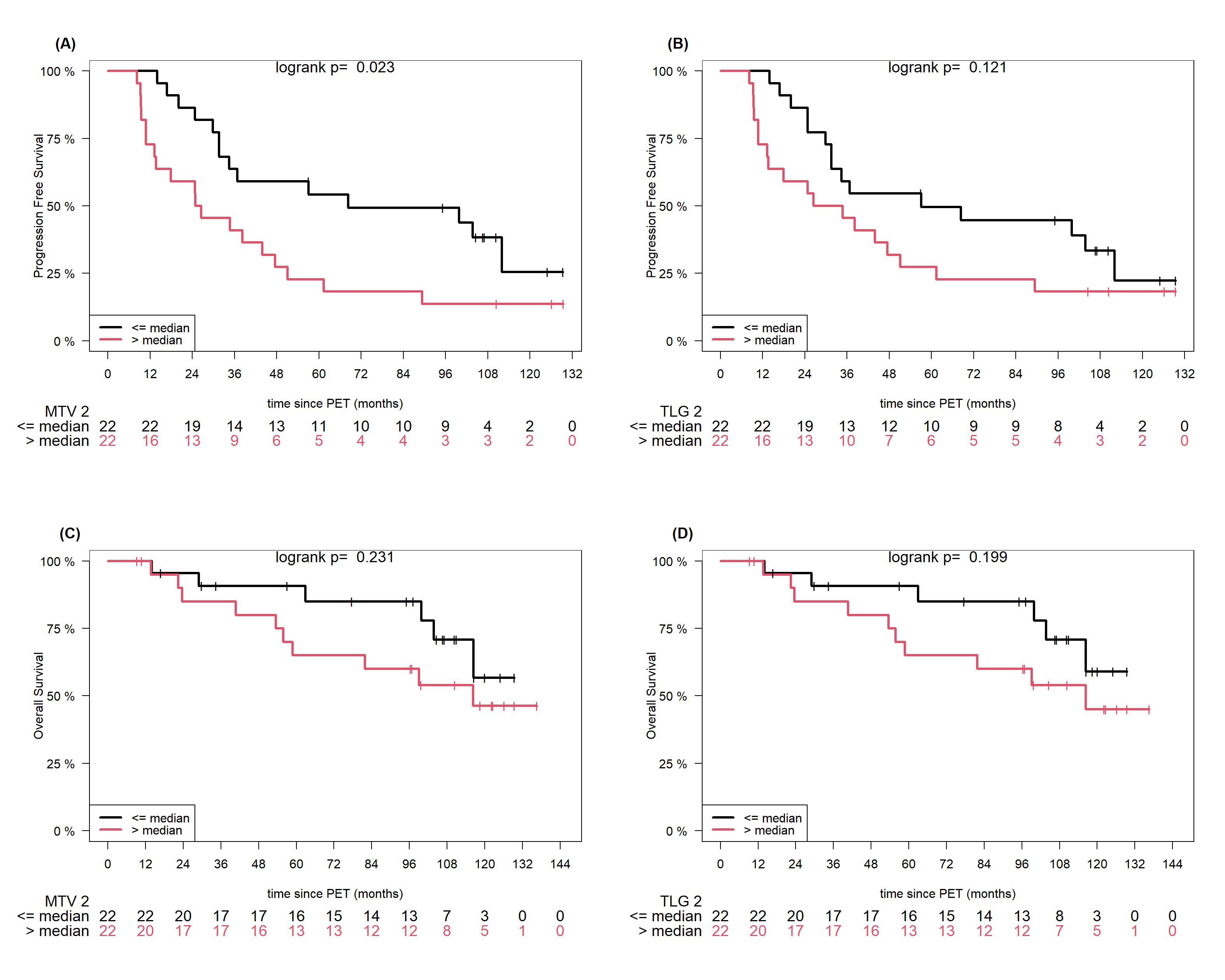


**Supplementary Figure 3** Kaplan–Meier estimates of PFS according to AI-derived, whole-body MTV (A) and TLG (B) as well as estimates of OS according to whole-body MTV (C) and TLG (D), based on approach 3. The numbers of patients at risk in each group and for the respective time points are shown below the plots.


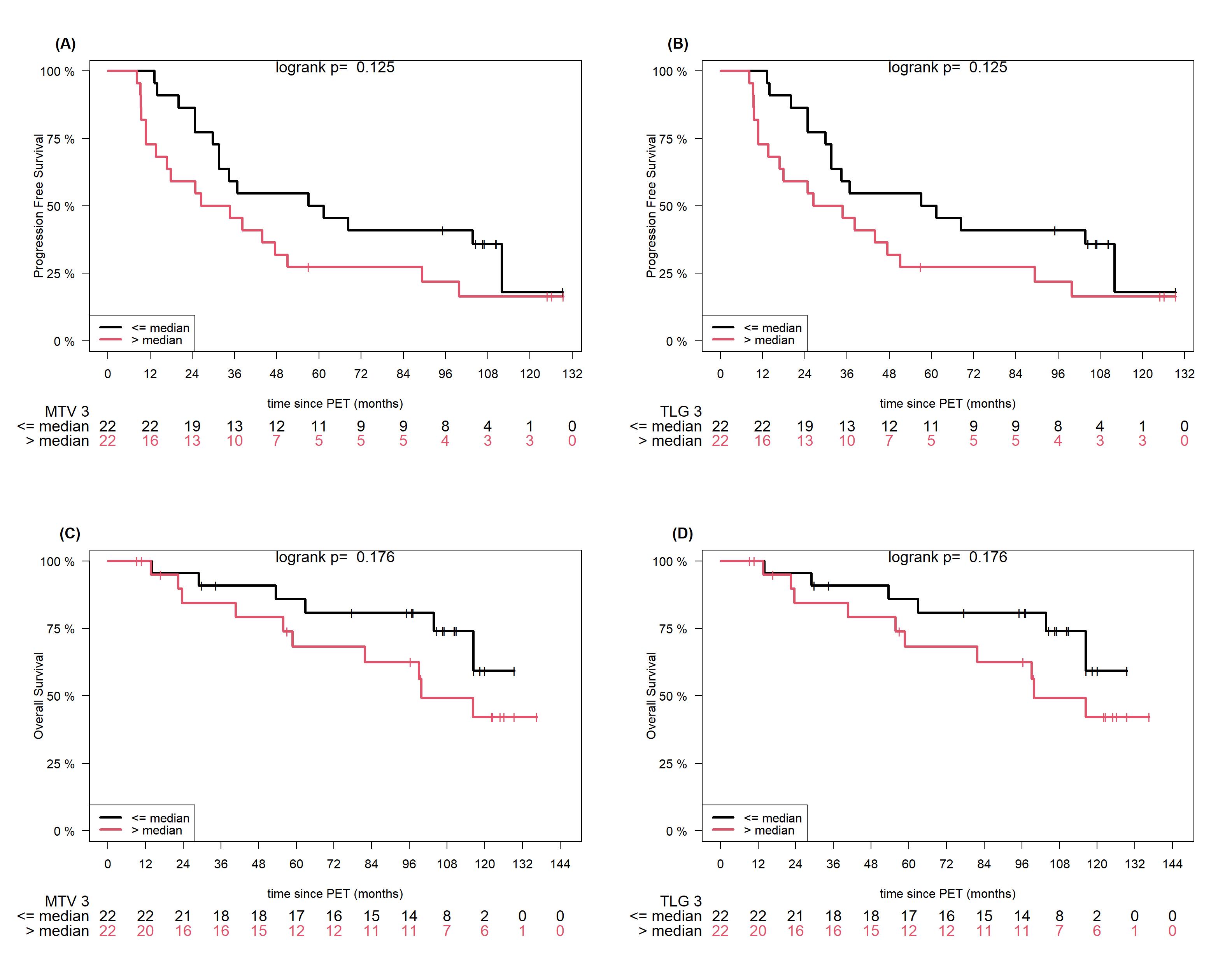


**Supplementary Figure 4** Kaplan–Meier estimates of PFS according to AI-derived, whole-body MTV (A) and TLG (B) as well as estimates of OS according to whole-body MTV (C) and TLG (D), based on approach 4. The numbers of patients at risk in each group and for the respective time points are shown below the plots.
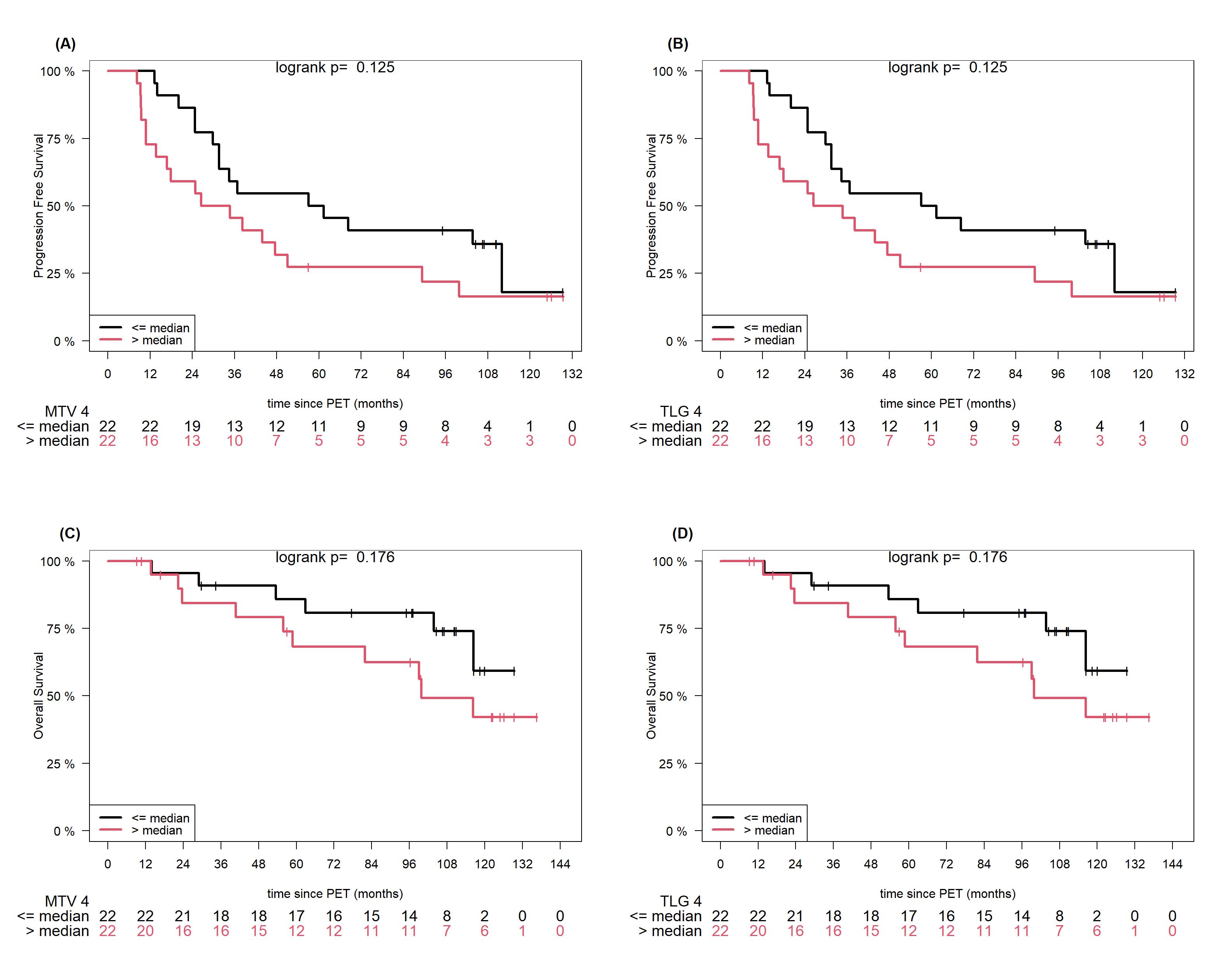


**Supplementary Figure 5** Kaplan–Meier estimates of PFS according to AI-derived, whole-body MTV (A) and TLG (B) as well as estimates of OS according to whole-body MTV (C) and TLG (D), based on approach 5. The numbers of patients at risk in each group and for the respective time points are shown below the plots.


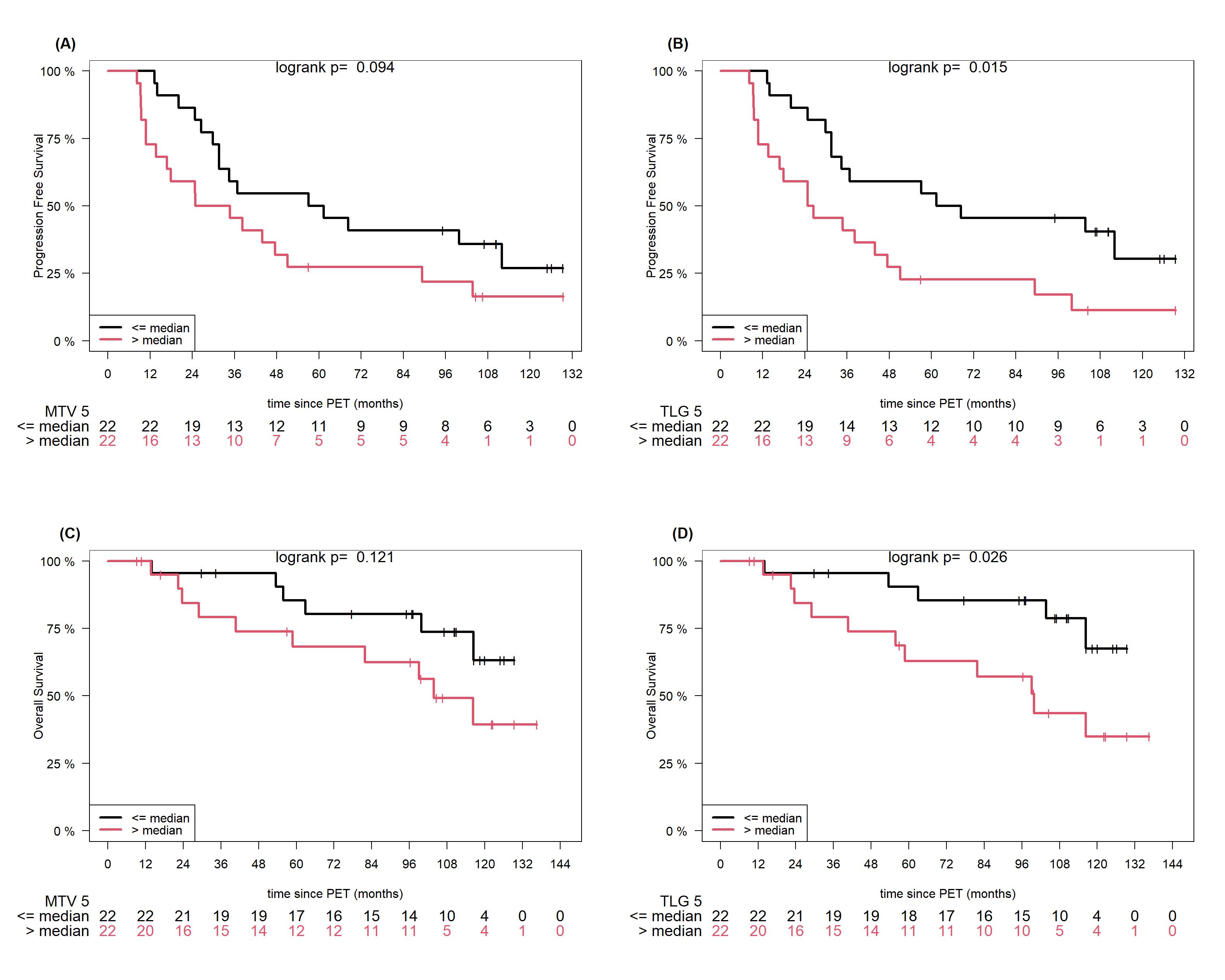


**Supplementary Figure 6** Kaplan–Meier estimates of PFS according to AI-derived, whole-body MTV (A) and TLG (B) as well as estimates of OS according to whole-body MTV (C) and TLG (D), based on approach 6. The numbers of patients at risk in each group and for the respective time points are shown below the plots.


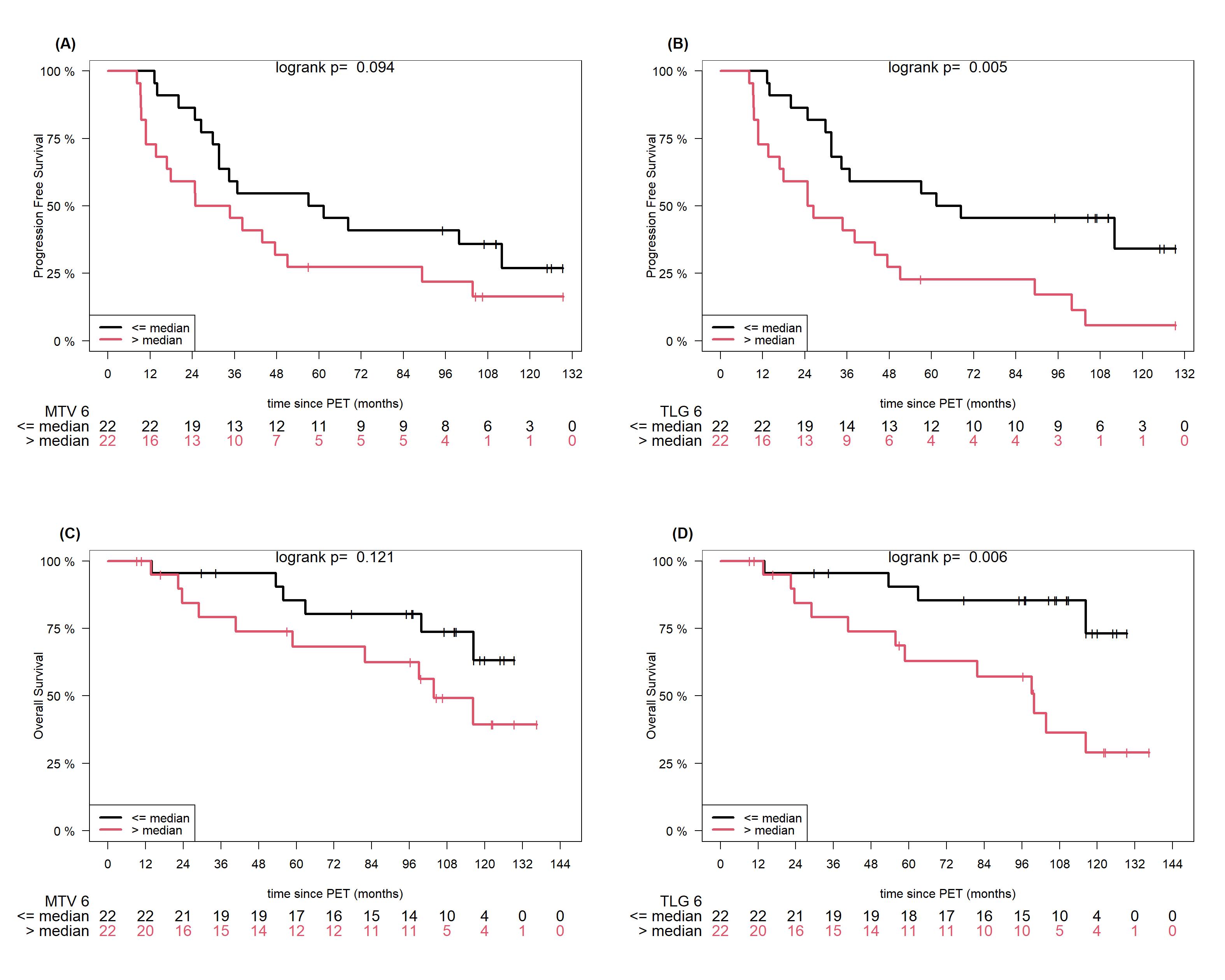


**Supplementary Figure 7** Kaplan–Meier estimates of PFS according to AI-derived, whole-body MTV (A) and TLG (B) as well as estimates of OS according to whole-body MTV (C) and TLG (D), based on approach 8. The numbers of patients at risk in each group and for the respective time points are shown below the plots.


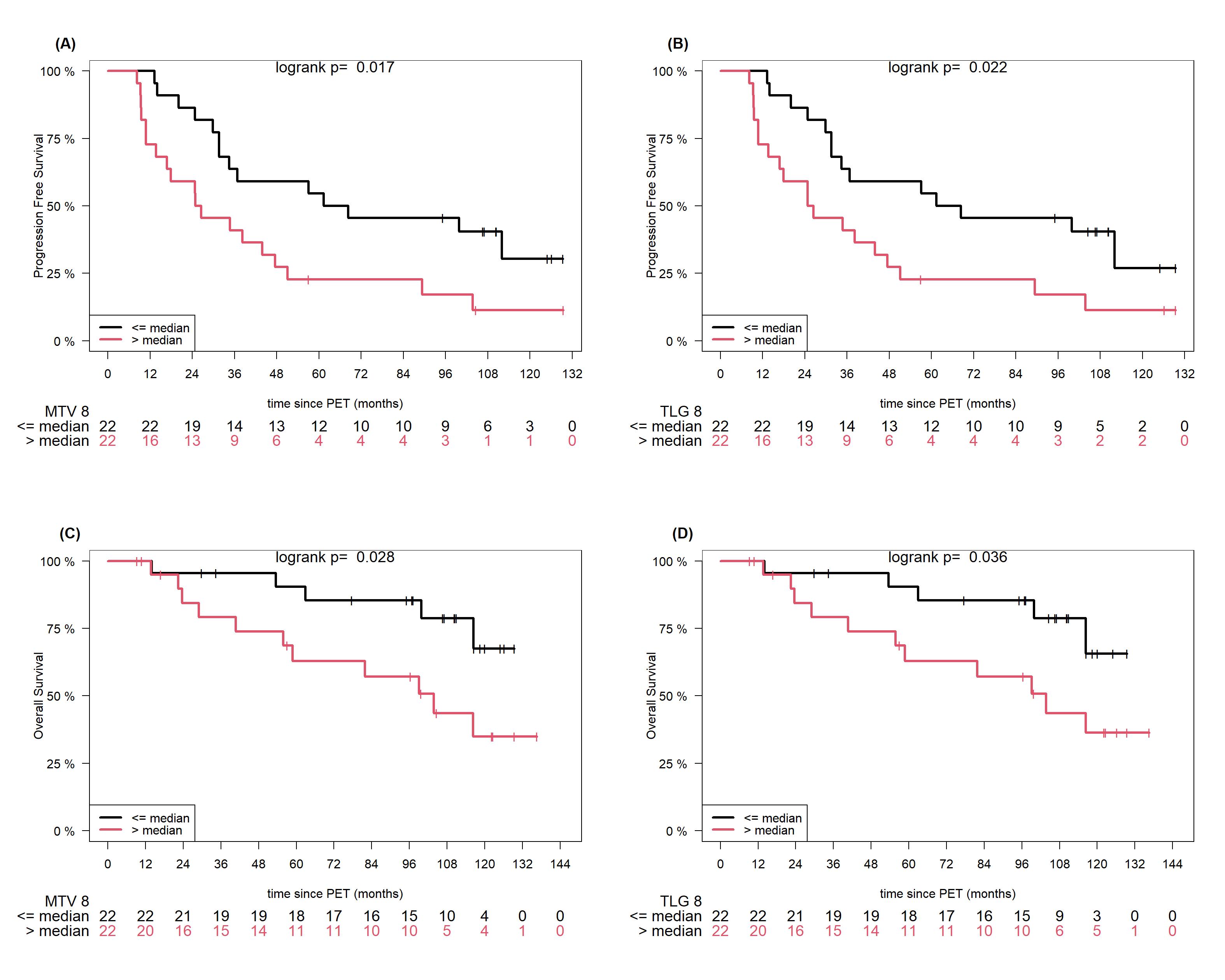


**Supplementary Figure 8** Kaplan–Meier estimates of PFS according to AI-derived, whole-body MTV (A) and TLG (B) as well as estimates of OS according to whole-body MTV (C) and TLG (D), based on approach 9. The numbers of patients at risk in each group and for the respective time points are shown below the plots.


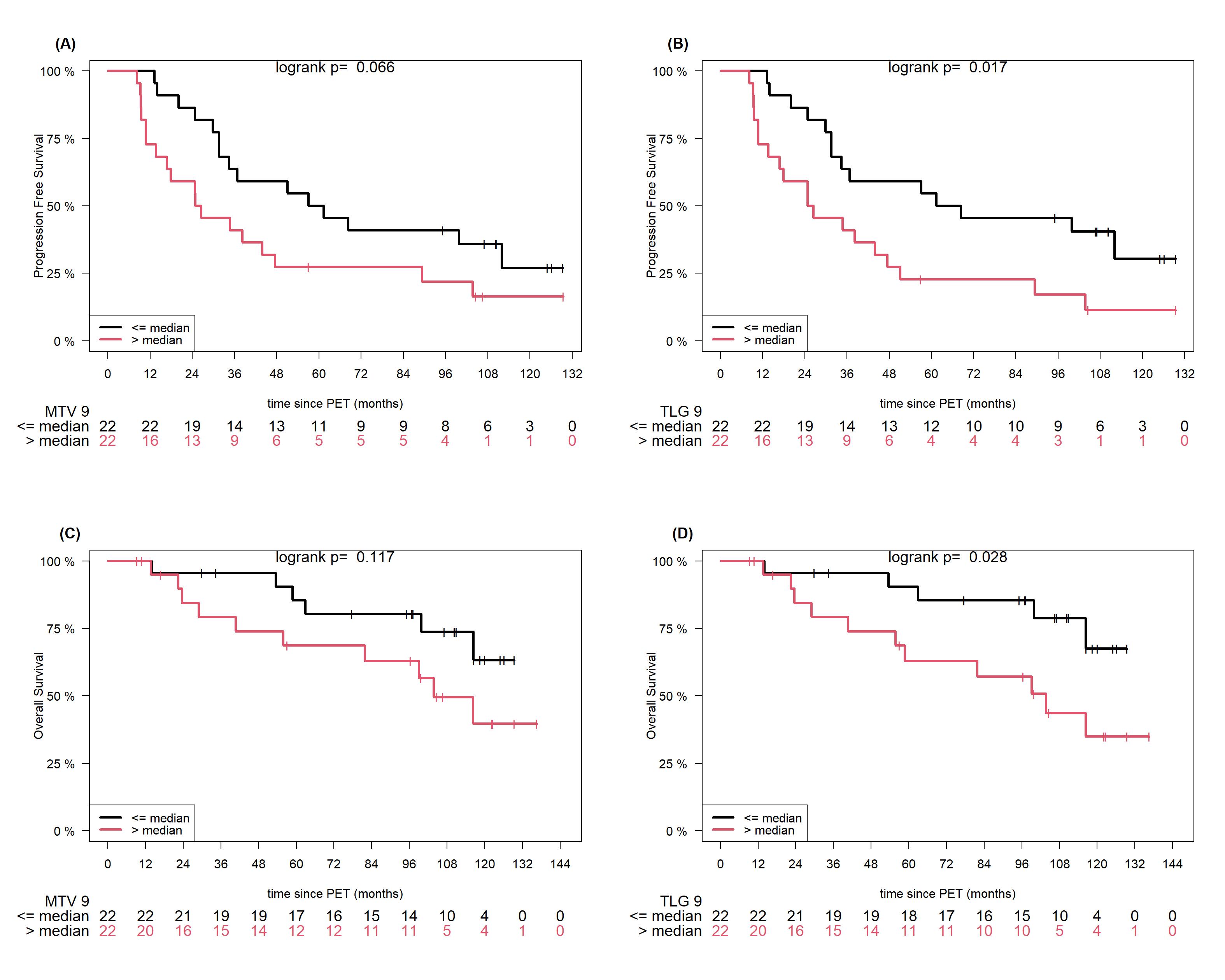


**Supplementary Figure 9** Kaplan–Meier estimates of PFS according to AI-derived, whole-body MTV (A) and TLG (B) as well as estimates of OS according to whole-body MTV (C) and TLG (D), based on approach 10. The numbers of patients at risk in each group and for the respective time points are shown below the plots.


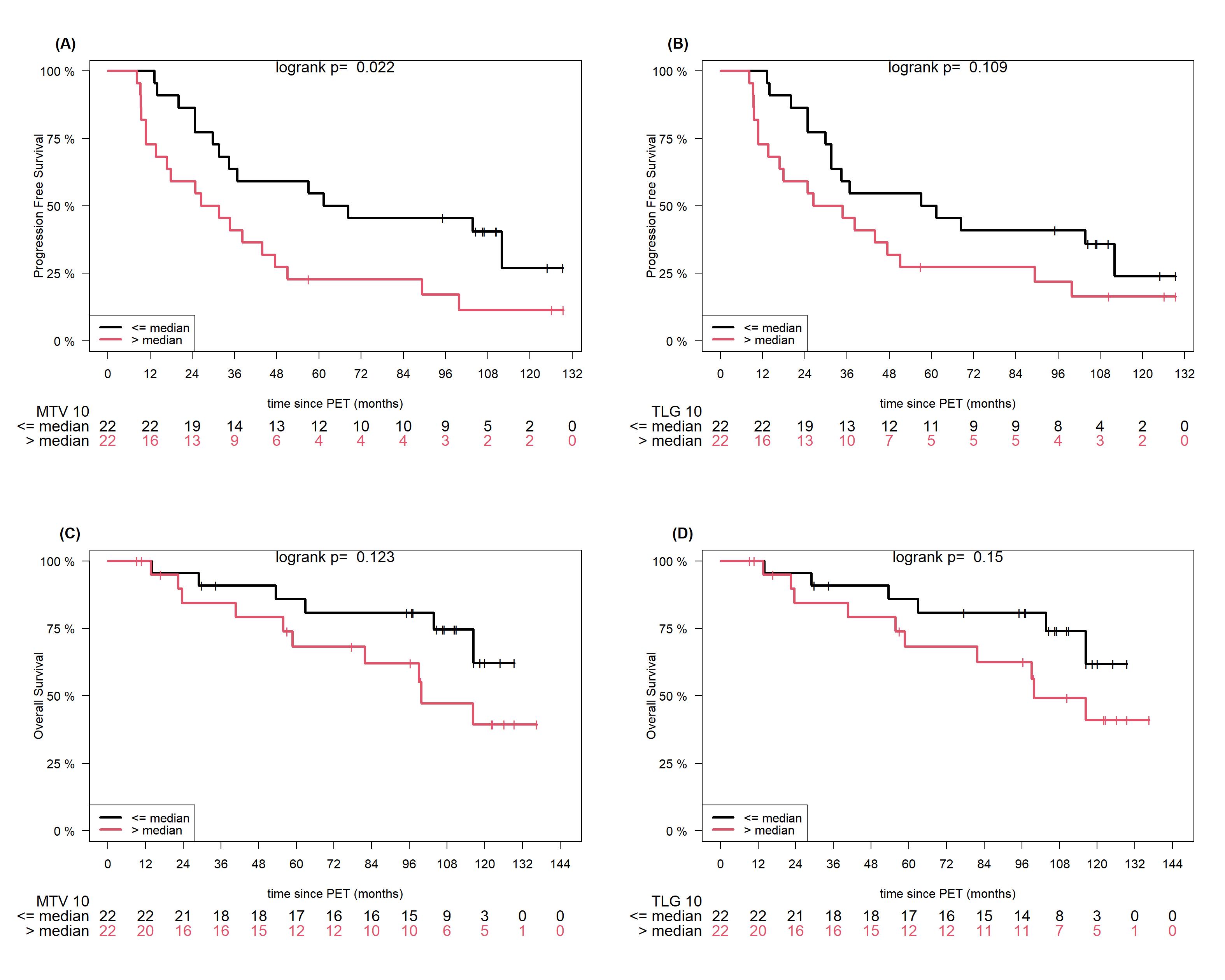

Supplement: Supplementary file 1 — Supplementary file1 (DOCX 3.47 KB) [file 259_2024_6668_MOESM1_ESM.docx]
